# Comparative metagenomics of microbial communities and resistome in southern farming systems: implications for antimicrobial stewardship and public health

**DOI:** 10.3389/fmicb.2024.1443292

**Published:** 2024-11-26

**Authors:** Agnes Kilonzo-Nthenge, Iftekhar Rafiqullah, Michael Netherland, Maureen Nzomo, Abdullah Mafiz, Samuel Nahashon, Nur A. Hasan

**Affiliations:** ^1^Department of Food and Animal Sciences, Tennessee State University, Nashville, TN, United States; ^2^EzBiome Inc., Gaithersburg, MD, United States; ^3^Center for Bioinformatics and Computational Biology, University of Maryland, College Park, MD, United States

**Keywords:** microbial community, antimicrobial resistance, cattle and poultry farming, Alabama, Tennessee, food safety

## Abstract

Agricultural practices significantly influence microbial diversity and the distribution of virulence and antimicrobial resistance (AMR) genes, with implications for ecosystem health and food safety. This study used metagenomic sequencing to analyze 60 samples (30 per state) including water, soil, and manure (10 each) from Alabama (a mix of cattle and poultry sources) and Tennessee (primarily from cattle). The results highlighted a rich microbial diversity, predominantly comprising Bacteria (67%) and Viruses (33%), with a total of over 1,950 microbial species identified. The dominant bacterial phyla were Proteobacteria, Cyanobacteria, Actinobacteria, Firmicutes, and Bacteroidetes, with the viral communities primarily represented by Phixviricota and Uroviricota. Distinct state-specific microbial profiles were evident, with Alabama demonstrating a higher prevalence of viral populations and unique bacterial phyla compared to Tennessee. The influence of environmental and agricultural practices was reflected in the microbial compositions: soil samples were notably rich in Actinobacteria, water samples were dominated by Proteobacteria and Cyanobacteria, and manure samples from Alabama showed a predominance of Actinobacteria. Further analyses, including diversity assessment and enterotype clustering, revealed complex microbial structures. Tennessee showed higher microbial diversity and phylogenetic complexity across most sample types compared to Alabama, with poultry-related samples displaying distinct diversity trends. Principal Coordinate Analysis (PCoA) highlighted notable state-specific variations, particularly in manure samples. Differential abundance analysis demonstrated elevated levels of *Deinococcus* and *Ligilactobacillus* in Alabama, indicating regional effects on microbial distributions. The virulome analysis revealed a significant presence of virulence genes in samples from Alabama. The community resistome was extensive, encompassing 109 AMR genes across 18 antibiotic classes, with manure samples displaying considerable diversity. Ecological analysis of the interactions between AMR gene subtypes and microbial taxa revealed a sophisticated network, often facilitated by bacteriophages. These findings underscore the critical role of agricultural practices in shaping microbial diversity and resistance patterns, highlighting the need for targeted AMR mitigation strategies in agricultural ecosystems to protect both public health and environmental integrity.

## Introduction

Antimicrobial resistance poses a grave and urgent threat to global public health in the 21st century. The World Health Organization (WHO) has identified it as one of the foremost challenges of our time (WHO, 2023). Projections suggest that by 2050, antimicrobial resistance could lead to an alarming 10 million deaths annually, with an estimated cumulative economic burden exceeding $100 trillion (Tang et al., 2017). While antimicrobial resistance is a natural phenomenon, its acceleration and spread are exacerbated by multifaceted pathways, through which pathogenic bacteria can acquire resistance and disseminate resistance between animals and humans (Graham et al., 2019; Machalaba et al., 2015). These transmission pathways include the consumption of animal-derived foods, the use of manure as fertilizer, direct contact with farm animals, contamination of water bodies through surface run-offs carrying fecal matter, and soil contamination by antibiotic residues from animal waste products, among others. Previous studies have shown that a substantial proportion, ranging from 30 to 90% of veterinary antibiotics are released into the environment as unmetabolized waste products (Graham et al., 2019; Machalaba et al., 2015). Additionally, occupational exposures among farmworkers have been linked to an increased prevalence of antibiotic-resistance *Escherichia coli* in their guts microbiota (Graham et al., 2019; Machalaba et al., 2015).

Notably, antimicrobial-resistant bacteria possess the capacity to spread across diverse ecosystems (Li et al., 2023; Ma et al., 2021; Rasheed et al., 2014). The major components of the agroecosystem - animals, animal products, manure, soil, and water- serve as reservoirs of both pathogenic and commensal bacteria, particularly antibiotic-resistant strains (Ma et al., 2021; Pandey et al., 2014; Miller et al., 2022). The use of antibiotics in food-producing animals has emerged as a significant contributor to the ongoing emergence and progression of antibiotic resistance in humans (Mann et al., 2021; Economou and Gousia, 2015). Despite extensive research exploring the relationship between antimicrobial use in animal production and human infections, there remains a scarcity of data on the prevalence and spatial distribution of antimicrobial resistance determinants and antimicrobial-resistant foodborne pathogens and commensal bacteria in small- and medium-sized poultry and cattle farms. On the other hand, the microbial community within agricultural ecosystems plays a crucial role in shaping antibiotic resistance dynamics, influencing the emergence, spread, and persistence of resistant bacteria. As noted by Allen et al. (2010), microbial communities exhibit intricate interactions that can facilitate the horizontal transfer of antimicrobial resistance (AMR) genes among diverse bacterial taxa. This horizontal gene transfer, occurring within the complex network of microbial populations, contributes significantly to the dissemination of antibiotic resistance in agricultural settings (Martínez, 2008). Moreover, the composition and diversity of microbial communities can impact the prevalence of antibiotic resistance. Studies have shown that diverse microbial communities tend to harbor a greater diversity of ARGs (Forsberg et al., 2014). The presence of certain bacterial species within the microbiome may also influence the abundance and distribution of ARGs. For example, commensal bacteria such as *Escherichia coli* and *Enterococcus* spp. have been implicated as reservoirs of AMR genes in agricultural environments (Durso et al., 2012; Karkman et al., 2016).

Additionally, microbial communities can act as hotspots for the co-selection of antibiotic resistance traits (Seiler and Berendonk, 2012). Co-selection occurs when the use of one antibiotic selects for resistance to multiple antibiotics or other stressors, leading to the enrichment of multidrug-resistant bacteria (Wellington et al., 2013). This phenomenon is particularly relevant in agricultural settings, where the widespread use of antibiotics in livestock production can exert selective pressure on microbial communities, promoting the proliferation of antibiotic-resistant strains (Gaze et al., 2013). Furthermore, the spatial distribution of microbial communities within farming systems can influence the dissemination of antibiotic resistance. Variations in microbial community structure and composition between different ecological niches, such as soil, manure, and water, can impact the transfer of AMR genes and resistant bacteria within the agricultural environment (Wang et al., 2014).

Recent research has highlighted that soil serves as a critical hotspot for antibiotic resistance, where antibiotic residues and resistant bacteria can accumulate and propagate resistance genes within microbial communities. Studies have elucidated the interactions between antibiotic residues in soil and the emergence of antimicrobial resistance genes, as well as the environmental impact of manure application on soil microbial ecosystems (Wang et al., 2015; Bao et al., 2024). The persistence of antibiotic residues in agricultural soil has been shown to promote horizontal gene transfer, which enhances the spread of AMR genes across bacterial species (Han et al., 2022; Baquero et al., 2022). Additionally, soil bacteria, including both pathogenic and commensal organisms, play a pivotal role in the transfer of these resistance genes across ecosystems, posing a significant risk to human health (Wang et al., 2023; Selvarajan et al., 2022).

In summary, microbial communities play a multifaceted role in antibiotic resistance dynamics within agricultural ecosystems. Understanding the complex interactions between microbial populations, AMR genes, and environmental factors is essential for developing effective strategies to mitigate the spread of antibiotic resistance in food production systems. Given the varying impact of agricultural practices on microbial dynamics and antimicrobial resistance, this study hypothesizes that distinct farming systems and environmental conditions in Alabama and Tennessee differentially shape the microbial communities and resistome in southern farming ecosystems. Through comparative metagenomic analysis, we aim to assess how variations in farming practices influence the distribution of microbial species, virulence factors, and antimicrobial resistance genes across soil, manure, and water samples. The study further hypothesizes that these factors have significant implications for antimicrobial stewardship and public health by identifying key microbial taxa that act as reservoirs for antibiotic-resistant genes. This study will provide insights into how agricultural systems contribute to the spread of antimicrobial resistance and inform targeted AMR mitigation strategies for more sustainable farming practices and enhanced public health outcomes (Table 1).

**Table 1:** Distribution of samples collected from various sources and farming systems from two southern states (*N* = 60).

State/Sample type	Alabama (*n* = 30)			Tennessee (*n* = 30)		
	Cattle	Poultry	Sub-total	Cattle	Poultry	Sub-total
Manure	7	3	10	10	0	10
Soil	10	0	10	8	2	10
Water	8	2	10	10	0	10
Total	25	5	30	28	2	30

## Materials and methods

### Study sites and sample collection

The environmental samples were collected from six poultry farms (1 in Tennessee and 5 in Alabama) and 31 cattle farms (16 in Tennessee and 15 in Alabama) between September 2019 and August 2020 through collaboration with Tennessee State University (TN) and Tuskegee University (AL) Extension Programs. All sampling sites experienced a humid subtropical climate, with Tennessee sites receiving 50–55 inches (1270–1,400 mm) of rainfall annually and Alabama sites receiving 50–60 inches (1270–1,520 mm). These are generally small-scale farms, with incomes under $350,000 as reported by the USDA, and an average size of 231 acres. Soil samples were collected from three designated sites on each farm (S1, S2, and S3), spaced 50 meters apart, with triplicate samples of 200 grams collected from a depth of 5–10 cm at each site. Manure samples were collected from three sites (M1, M2, and M3) at each farm. Three samples were aseptically taken from solid manure piles less than 2 weeks old, creating composite samples of 500 grams. Water samples were collected from three sites (W1, W2, and W3) at each farm, including feeding areas and nearby water bodies such as rivers, lakes, ponds, or streams. Sterile plastic bottles were submerged below the water surface for collection and sealed to prevent contamination. A simple randomization of sample collection was used for collecting oil, manure, and water samples to eliminate systematic errors and ensure that the selection process was fair and unbiased. Soil and manure samples collection was done using disposable plastic spoons and transferred into sterile sampling bags (Fisher brand, Pittsburg, PA). Water samples were collected in 500 mL sterile bottles from each farm. All samples were collected in triplicates per site and labeled with the farm identification number and the dates of collection. Immediately after collection, samples were transported using coolers (≈ 4°C) and subsequently stored at −80°C till analysis. Water samples were collected from three sites in each cattle and poultry farms from feeding areas and any other nearby water bodies such as a river, lake, pond, and a stream. A bottle was filled by plunging downwards below the water surface and resealed tightly to avoid leakage. Soil samples were collected in triplicates of 200 g from each site. The distance between the three sites was 50 m apart and the collection was done to a depth of 5–10 cm. The soil samples from each designated site were pooled into one composite sample of approximately 600 g. The soil was properly mixed and carefully cleaned of any extraneous materials such as debris, leaves, stems, and roots, using sterilized forceps to avoid contamination. All tools were sterilized, and gloves were changed between uses to maintain the integrity of the samples. Manure samples from three sites from each cattle and poultry facility manure were aseptically collected to make a composite sample of 500 g from solid manure from each pile using the simple random technique. The solid samples were collected from piles less than 2 weeks old and were termed as Fresh Pile. Samples were processed immediately upon arrival to the laboratory for standard microbiology analysis. An aliquot of samples was kept frozen in the −80°C for DNA extraction and metagenomic sequencing. All together a total of 60 samples (water, soil, and manure, 20 each) were collected from Alabama and Tennessee. Thirty samples were collected from each state to ensure representative sampling, from both cattle farming (53 samples) and poultry farming (*n* = 7 samples) facilities. Poultry sampling was significantly reduced during the COVID-19 pandemic due to restricted farm visits. Supplementary Table S1 demonstrates the detailed distribution of the sample types among two states and farming practices.

### DNA extraction

Frozen samples were thawed at room temperature and an aliquot of samples (250 mg manure and soil) were lysed and homogenized in a bead beating tube containing lysis buffer and 0.1 mm and 0.5 mm bashing beads using Omni’s Bead Ruptor 96 (Perkin Elmer, United States) as per manufacturer instructions. Total DNA was then isolated using ZymoBiomics DNA extraction kit (Zymo Research, USA) with a final elution volume of 50 μL. Three water subsamples (500 mL/each) from the same farm were also mixed, and then concentrated each 500 mL water by filtering through a sterile disposable vacuum filter unit with 0.22 μm polyethersulfone (PES) membranes (Fisher Scientific, Pittsburgh, PA). PowerWater® DNA Isolation Kit (MO BIO Laboratories, Inc., Carlsbad, CA) was used to extract DNA. Concentration of genomic dsDNA was measured using Qubit® 4.0 Fluorometer (Thermo Fisher Scientific, Waltham, MA, United States).

### Library preparation and shotgun metagenomic sequencing

Metagenomics sequencing was performed at EzBiome Inc. (Gaithersburg, MD, United States). 50 ng-1ug of genomic DNA was used for library construction using NEB Next® Ultra™ II FS DNA Library Prep Kit for Illumina. Briefly, gDNA was enzymatically sheared, DNA fragment ends were repaired, 3′ adenylated, and ligated to adapters according to the manufacturer’s instructions. The resulting adapter-ligated libraries were PCR-amplified using an initial denaturation step performed at 98°C for 45 s followed by 5 cycles of denaturation (98°C, 15 s), annealing (60°C, 30 s) and extension (72°C, 30 s), and a final elongation of 1 min at 72°C. PCR products were cleaned up with magnetic beads. The libraries were then quantified and qualified using the Agilent 2,200 TapeStation instrument followed by normalization and multiplexed sequencing on HiseqX10 sequencer (Illumina, San Diego, CA, United States) using the pair-end 150 bp run format. All samples successfully underwent metagenomic shotgun sequencing, yielding a total of 1.25 billion raw reads. On average, each sample yielded 20.77 million raw reads, ranging from 8.4 million to 38.25 million. Supplementary Figure S1 illustrates the distribution of reads in each sequenced sample.

### Metagenomic taxonomic and functional profiling

Taxonomic and functional profiling of the datasets were conducted as described by Bhattacharjee et al. (2024), Brumfield et al. (2023), and Ibekwe et al. (2023). Briefly, Kraken2 (Wood et al., 2019) with a pre-built core gene database (Chalita et al., 2020) containing k-mers (*k* = 35) of reference genomes from the EzBioCloud database (Yoon et al., 2017) was used to identify bacterial and archaeal species. Fungal and viral genomes from NCBI refseq database were added to the Kraken2 database. A custom bowtie2 (Langmead and Salzberg, 2012) database was built from identified species, and raw samples were mapped using the --very-sensitive option and a quality threshold of phred33. Samtools (Li et al., 2009) and Bedtools (Quinlan and Hall, 2010) were used to process the output. Species were quantified if core genes or genomes had at least 25% coverage. Finally, species abundance was calculated using the total number of reads counted and normalized species abundance was calculated by using the total length of all their references. DIAMOND (Buchfink et al., 2015) was used for functional annotation against the KEGG database (Kanehisa et al., 2017) using the blastx parameter. If a read had multiple KEGG hits, the top hit was always used. After quantifying the KEGG orthologs, minpath (Ye and Doak, 2009) was used to predict the presence of KEGG functional pathways.

### Comparative metagenomic and statistical analyses

Various comparative metagenomics and statistical methods were used to compare microbiome composition, diversity, resistome, and virulome across different sample types, farming practices, and states. For microbiome composition analysis, average relative abundances per genus per sample type were calculated and visualized using sunburst charts. Alpha diversity was measured using Shannon Diversity (Shannon, 1948) and Faith’s phylogenetic diversity (PD) (Faith, 1992) on the EzBioCloudPro platform (https://www.ezbiocloudpro.app/). Enterotype analysis was performed on genus relative abundance as described by Arumugam et al. (2011). PAM clustering (Kaufman and Rousseeuw, 1990) and the Calinski-Harabasz (CH) Index (Caliński and Harabasz, 1974) determined optimal clusters. Beta diversity was analyzed using pairwise Bray-Curtis distances, followed by Principal Coordinate Analysis (PCoA). Pairwise PERMANOVA analyses determined statistical differences between sample groups in PCoA ordination.

### Differential abundance

Differential abundance analysis (DAA) identifies candidate biomarkers for the group of interest. Seven different DAA methods available through EzBioCloudPro Platform which comprises: DESeq2, LinDA, LEfSe, ANCOM-BC, ALDEx2, MaAsLin2, and SIAMCAT (Love et al., 2014; Zhou et al., 2022; Segata et al., 2011; Lin and Peddada, 2020; Fernandes et al., 2013; Mallick et al., 2021; Wirbel et al., 2021, respectively) were employed. The results of each DAA tool were compared to find consensus biomarkers.

### Antimicrobial resistance (AMR) and virulence factor (VF) gene profiling

Antibiotic resistance gene (ARG) profiles were produced using a pre-built bowtie2 (Langmead and Salzberg, 2012) database from NCBI’s NDARO (www.ncbi.nlm.nih.gov/pathogens/antimicrobial-resistance/) reference genes. The analysis focused on identifying acquired antibiotic resistance genes (ARGs) derived from metagenomic sequence data. Intrinsic resistance mechanisms, which are naturally occurring and not typically associated with horizontal gene transfer, were not the focus of this study. Virulence factor (VF) profiles were generated using a pre-built bowtie2 database from the VFDB (http://www.mgc.ac.cn/VFs/), covering VFs from 32 bacterial genera (Liu et al., 2019). Each metagenome read was mapped against these genes using bowtie2 with the --very-sensitive option, and processed using samtools (Li et al., 2009). De-duplication was performed with a custom script selecting the highest coverage alignment for each gene. Data subsets were created with cut-offs of >40 and > 70% reference gene coverage. Further datasets for AMR gene class, VF gene reference, and VF gene category were generated by summing shared classes, references, and categories within each sample and visualized with Sankey Graphs to demonstrate the distribution and flow of VF genes across categories and potential hosts.

### Ecological analysis

Envfit and co-occurrence analyses were used to gauge potential associations of AMR genes with sample types and microbial taxa. The R package envfit was employed to uncover significant associations of AMR gene classes with sample types. Genus read counts were normalized using DESeq2’s median of ratios method (Love et al., 2014), and a dissimilarity matrix was created using vegdist in the vegan R package for PCoA via cmdscale. The resulting data frame was used for envfit analysis along with base abundances for gene sets with >40% reference coverage, summed by AMR class as “environmental vectors.” To visualize correlations, a correlation matrix was constructed by calculating all possible pairwise Spearman’s rank correlations between the 84 ARG subtypes found in the environmental samples (Steele et al., 2011). A correlation was considered statistically robust if Spearman’s correlation coefficient (*ρ*) was >0.8 and *p*-value <0.01 (Junker and Schreiber, 2008). *p*-values were adjusted using the Benjamini–Hochberg method to reduce false positives (Benjamini and Hochberg, 1995). Robust pairwise correlations of the ARG subtypes formed co-occurrence networks, analyzed in R using VEGAN v2.6–4 and igraph v1.4.1, with network visualization conducted on Gephi v0.10.1.

### Statistical analyses

Alpha diversity statistics were calculated using the ranksum function and Spearman correlation coefficient statistics were calculated using the ‘spearmanr’ funciton from the Python package scipy. PERMANOVA statistics were calculated using the vegan v2.6–4 R package.

## Results

### Demographic and operational insights on farming practices

The cattle and poultry farmers in Tennessee and Alabama show significant demographic trends: most farmers are older males with a high school education or less (TN: 79.3% male, 69% high school; AL: 92.3% male, 42.3% high school), with low percentages of female and young farmers under 35 years old (TN: 3.4%, AL: 3.8%). Antibiotic usage is mainly for therapeutic and prophylactic purposes, with a high percentage of farmers in Tennessee (82.4%) and Alabama (92.4% combined) using them accordingly. Record-keeping on antibiotic use is robust (TN: 82.8%, AL: 57.7%), surpassing previous reports, and most farmers consult veterinarians (TN: 86.2%, AL: 76.9%). However, awareness of AMR as a public health issue remains low (TN: 65.4%, AL: 69%), indicating a need for better educational efforts. Manure management varies; Alabama predominantly uses stockpiling, while Tennessee employs a mix of methods including stockpiling (72.4%) and composting (20.7%). Dead animal disposal also differs, with Alabama entirely using dead animal services, whereas Tennessee uses multiple methods, such as deep burial and composting.

### Overall microbiome composition

**Figure 1:**
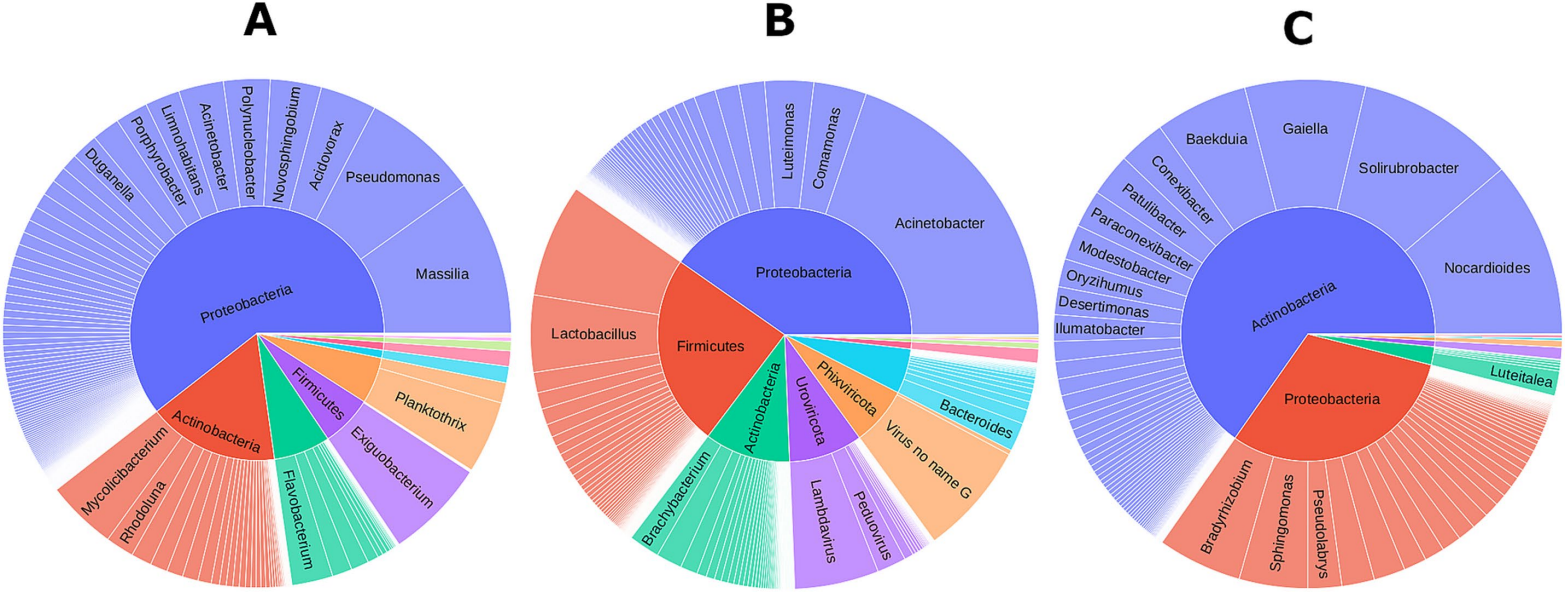
Microbial community associated with different farming systems across two states. **(A)** Overall composition of microbial communities in this study cohort; Microbiome community associated with Alabama **(B)**, and Tennessee **(C)** samples.

The microbial community across diverse agroecosystems and farming practices in Alabama and Tennessee was dominated by Bacteria (67.01%) and Viruses (32.85%), with minimal Archaea (0.09%) and Fungi (0.05%) (Supplementary Table S1). The bacterial diversity was remarkable, including over 1800 species from 448 genera, 155 families, and 12 phyla (Supplementary Tables S1, S2). Dominant phyla were Proteobacteria (30.75%), Cyanobacteria (15.86%), Actinobacteria (13.77%), Firmicutes (4.28%), and Bacteroides (1.81%) (Supplementary Table S3). The viral microbiota included over 140 DNA viral species from 36 genera and four phyla, with Phixviricota (19.32%) and Uroviricota (13.51%) being the predominant (Figure 1A and Supplementary Table S3). The fungal community had four fungal species across four genera from Ascomycota and Basidiomycota (Supplementary Tables S1, S2). The Archaeal diversity inluded five species from *Methanobrevibacter* genus (Figure 1 and Supplementary Table S1). Overall, the ecosystem showcased a diverse microbial community with over 1950 species across Bacteria, Viruses, Fungi, and Archaea with no protists detected in any of the samples analyzed.

Comparing microbiomes between Alabama and Tennessee, 8 out of 12 bacterial phyla were common in both states. Tennessee lacked Deinococcus and Verrucomicrobia, instead had Armatimonatedes and Tenericutes, which were absent in Alabama. In Alabama, Proteobacteria was most abundant (22.29%), followed by Actinobacteria (11.58%) and Firmicutes (4.14%) (Figure 1B and Supplementary Table S3). In Tennessee, Proteobacteria dominated (40.70%), followed by Cyanobacteria (34.25%) and Actinobacteria (16.34%) (Figure 1C and Supplementary Table S3). Interestingly, Alabama samples exhibited dominance of the viral community, mostly phages from Microviridae and Siphoviridae families, not observed in Tennessee. Microviridae comprises single-stranded DNA phages, while Siphoviridae includes lambda phages that infect bacteria.

### Microbial community across sample types and farming systems

**Figure 2:**
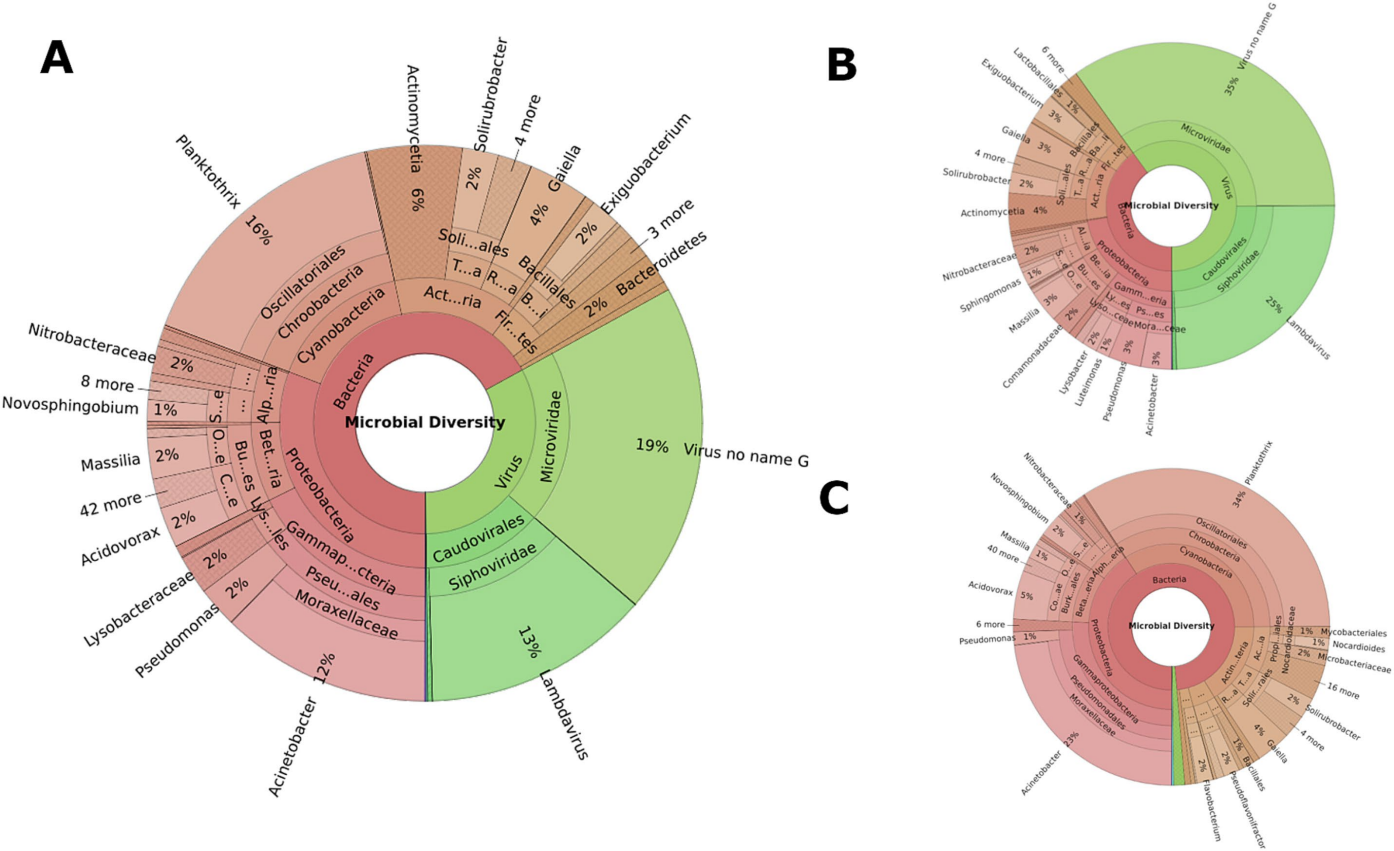
Overall composition of microbial community associated with different sample types. **(A–C)** represent water, manure, and soil samples, respectively. Inner circle represents phylum and outer circle represents genus level. Each phylum is represented by a distinct color with different shades of the same color representing distinct genera under each phylum.

Microbial composition analyses revealed distinct microbial landscapes across different sample types reflecting the ecological specificity of microbial communities and their adaptation to environmental niches (Figure 2). Soil samples were dominated by Actinobacteria (92.43%), with significant difference (*p* < 0.01), nearly double the abundance in manure (40.60%) and more than twice that in water (34.14%), highlighting their adaptation to terrestrial environments and vital role in soil ecology. Water samples demonstrated a predominance of Proteobacteria (46.74%), substantially higher than in manure (24.57%) and soil (6.69%), indicating an ecological preference for aquatic habitats (*p* < 0.01). Cyanobacteria were predominantly identified in water samples (7.86%) and absent in soil samples (Figure 2), reflecting their phototrophic nature.

**Figure 3:**
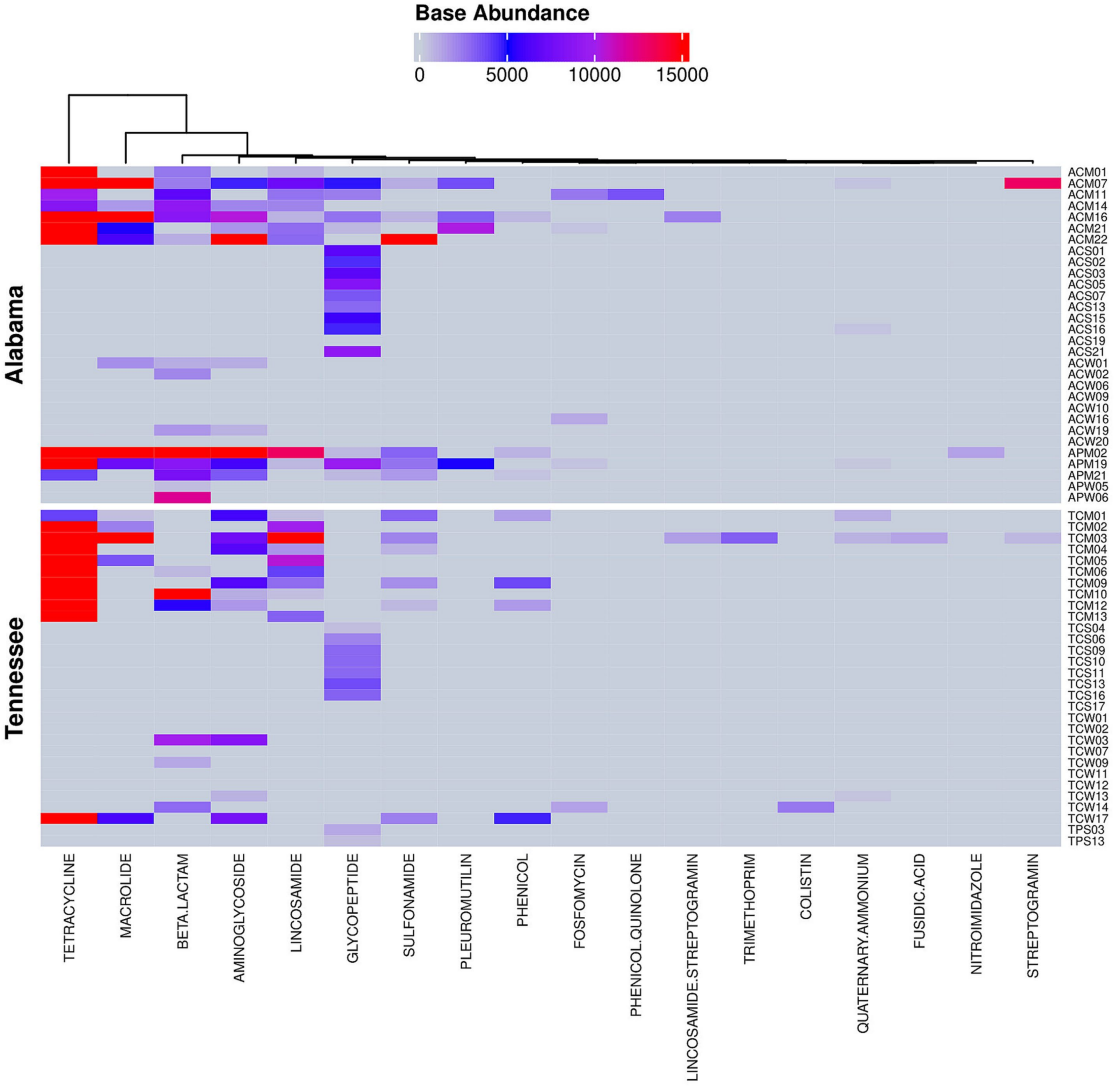
Interstate comparison of microbial communities associated with water, soil, and manure samples. In each of the five-digit alphanumeric numbers of samples where 1st digit represents state: “A” for Alabama, “T” for Tennessee; 2nd digit represents farming practices; “C” for Cattle, “P” for Poultry; and third digit represents sample types: “W” for Water; “S” for Soil and “M” for Manure, and finally numeric value represents the individual samples number. Different colors in the stacked bar represent relative abundance of distinct genera. Genera with >2% relative abundances were constituted as “others.”

Regionally, Tennessee water samples had a higher average abundance of Cyanobacteria (15.19%) compared to Alabama (0.52%), but this was not statistically significant (*p* > 0.05) (Figure 3). No significant differences were observed in soil samples between the states but discernible variations were observed in manure samples between states (Figure 3). Alabama manure samples had significantly higher Actinobacteria (64.58%) than Tennessee (16.61%, *p* = 0.03). Differences in other major bacterial phyla such Proteobacteria (13.95% vs. 35.19%), Firmicutes (11.46% vs. 31.15%), and Bacteroidetes (5.54% vs. 11.55%) between Alabama and Tennessee were not statistically significant (*p* > 0.05). No significant differences were observed between microbial compositions of cattle and poultry samples. However, the smaller number (*n* = 7) of poultry samples as opposed to the larger number (*n* = 53) of cattle samples limit the robustness of comparative conclusions.

### Microbial diversity

#### Alpha diversity

**Figure 4:**
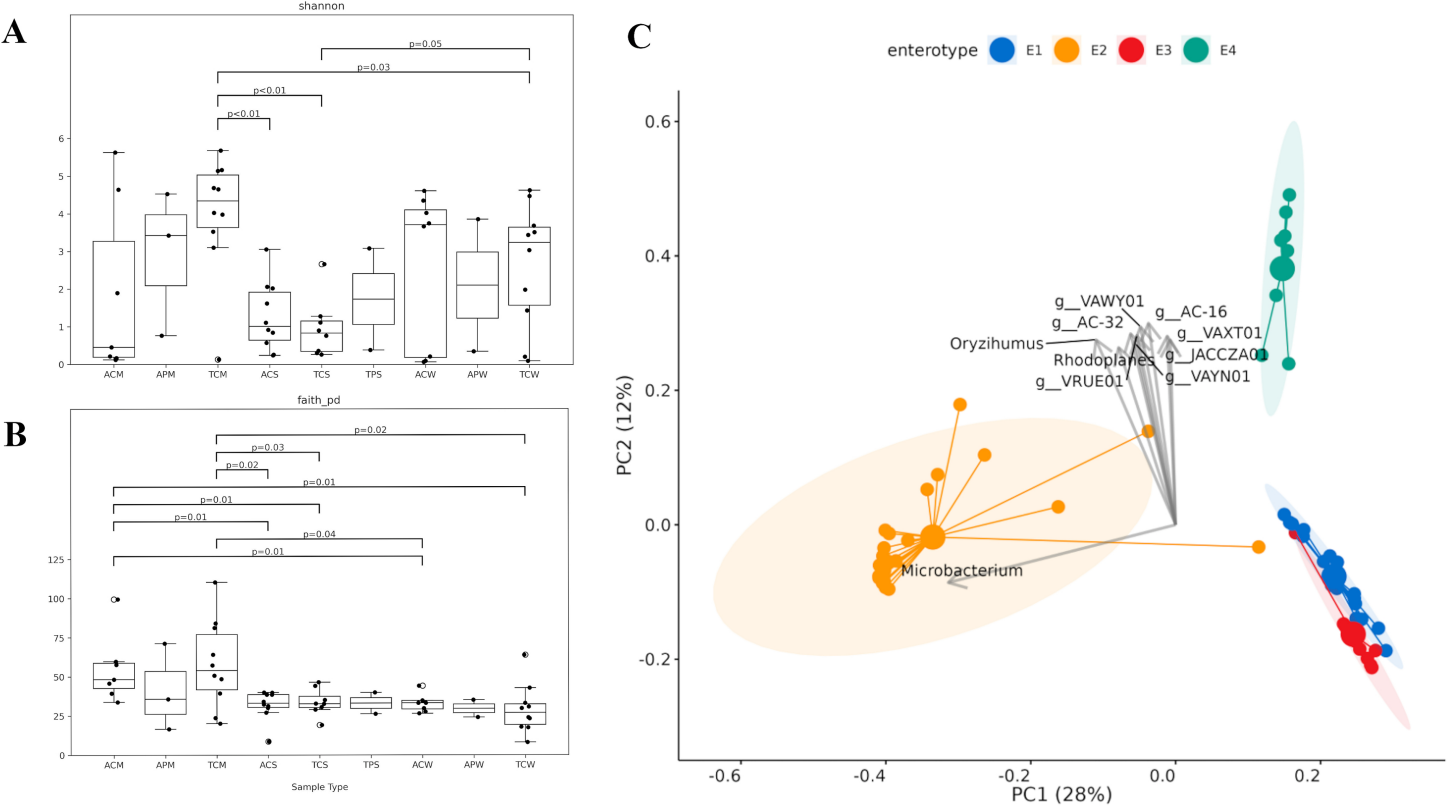
Observed species alpha diversity (richness and diversity) among different groups of samples. **(A)** Shannon diversity index; **(B)** Faith phylogenetic diversity; and **(C)** Overall enterotypes of all samples (PAM clustering). Connecting bars with *p*-values on top indicate the level of significance between the groups. Each enterotype is represented by a distinct color as shown in the figure.

The alpha diversity indices offer insights into the microbial richness, evenness, and the breadth of phylogenetic lineages within the microbial communities of each sample type. The observed Shannon diversity varied significantly across sample types and states (Figure 4A). In Alabama, cattle manure (ACM) exhibited a relatively low median index of 0.45, indicating a homogeneous microbial community (Supplementary Table S4), while cattle soil (ACS) displayed a slightly higher median index of 1.01, suggesting marginally richer microbial diversity. Water samples from cattle farms (ACW) showed the highest median Shannon index at 3.71, indicating a diverse microbial community. Poultry- samples displayed distinct trends, with poultry manure (APM) recording a higher median Shannon index of 3.42 compared to cattle manure, and poultry water (APW) had a median index of 1.62. In Tennessee, microbial diversity, especially in manure samples, surpassed that of Alabama. Cattle manure (TCM) exhibited a median Shannon index of 4.34, closely followed by cattle soil (TCS) with a median index of 4.32. Water samples from Tennessee cattle farms (TCW) also displayed high median Shannon indices (3.75). Poultry Soil (TPS) exhibited a median index of 1.74. Tennessee’s manure samples, both poultry and cattle, showed significantly higher diversity compared to soil and water samples.

Faith’s PD index, a measure of phylogenetic diversity, revealed distinct patterns across sample types and states (Figure 4B). In Tennessee, cattle manure (TCM) demonstrated the highest median phylogenetic diversity of 53.99, highlighting a rich and varied microbial ecosystem. Tennessee cattle soil (TCS) exhibited a median Faith’s PD of 32.72, comparable to Alabama’s cattle soil. Cattle water (TCW) samples had the lowest median PD among all Tennessee samples. Poultry soil (TPS) had a median PD of 33.12, consistent with cattle soil samples.

The parallel analysis of the Shannon diversity index and Faith’s PD across Alabama and Tennessee samples illuminates the intricate microbial landscape shaped by environmental and agricultural influences. Tennessee consistently showed elevated diversity and phylogenetic complexity compared to Alabama. Tennessee Cattle manure (TCM) exhibited the highest median values in both Shannon (4.34) and Faith’s PD (53.99), indicating abundant and evolutionarily diverse microbial communities. In contrast, Alabama had a more homogeneous microbial profile with lower Shannon diversity for cattle manure (ACM) but considerable phylogenetic depth. Alabama’s water samples exhibiting higher Shannon diversity compared to Tennessee, indicating nuanced microbial ecosystem differences. Poultry samples in both states exhibited intermediate levels of diversity and phylogenetic depth.

#### Enterotype

Enterotype analysis provides finer resolution in understanding the microbial community structure and its variations across different sample types. Enterotype clustering using the partitioning around medoids (PAM) method across all sixty (*n* = 60) samples from cattle and poultry farms in Alabama and Tennessee revealed intriguing patterns (Figure 4C). Overall, four distinct clusters were identified with the top two principal coordinate components explaining 40% of the variance at the genus level. Manure samples exhibited the highest variety with two clusters (E2, E3) where E2 was predominated by *Microbacterium*. Soil samples displayed fewer clusters (E1, E2, and E4), possibly due to the more stable and structured environment of the soil. Water samples showed an intermediate level of diversity, with clusters E1 and E2.

State-wise differences in cluster distributions were evident, with Alabama and Tennessee exhibiting distinct patterns. Both states shared some common clusters (E1, E2, and E4), but each also had unique clusters specific to its environmental conditions. Tennessee displayed a wider range of clusters, including E3, suggesting a greater microbial diversity compared to Alabama. Interestingly, the clustering patterns between cattle and poultry farming showed similarities, with both types sharing common clusters across the two states. However, poultry farming in Alabama appeared to have additional unique clusters (E2 and E4), indicating specific microbial compositions associated with this farming practice.

Further analysis of soil, water, and manure samples individually revealed distinct enterotypes compositions. Manure samples contained five enterotypes (E1-E5; Supplementary Figure S2A) with E1 predominantly comprising the genus *Microbacterium*, and E3 including *Bacteroides*. Tennessee manure contained all five enterotypes, while Alabama manures had four, lacking E5. Soil microbiota exhibited less diversity in terms of enterotypes, with only two types (E1 and E2; Supplementary Figure S2B) present in both Alabama and Tennessee where E1 was dominated by *Marmoricola*. Similarly, water samples displayed two enterotypes, both dominated by multiple genera (Supplementary Figure S2C).

#### Beta diversity

**Figure 5:**
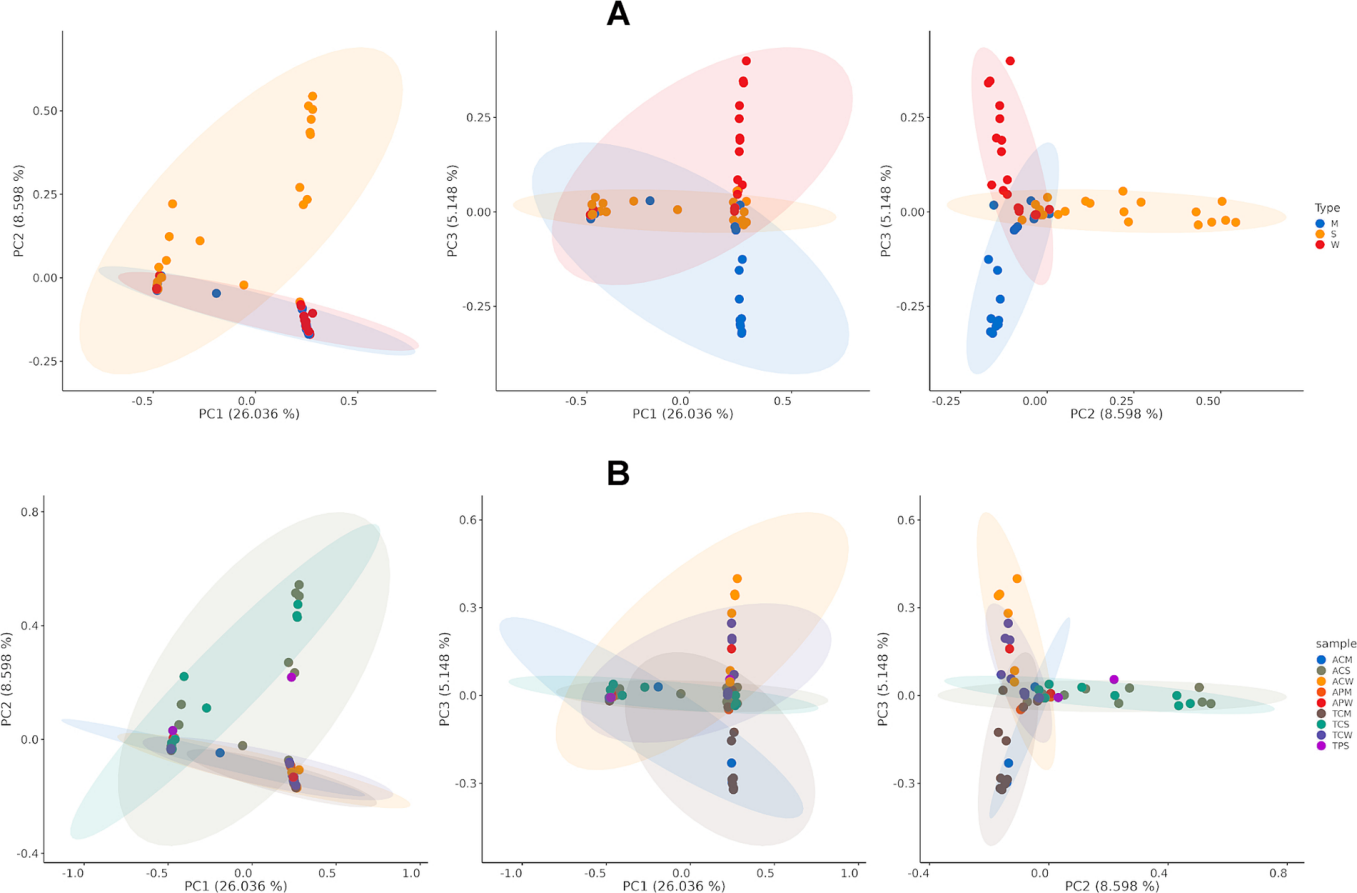
Principal coordinate analysis (PCoA) shows diversity dissimilarities of microbial composition among communities (beta diversity). **(A)** Beta diversity of soil, water, and manure samples. PERMANOVA *p*-value <0.05; and **(B)** Beta diversity across farm types and states. PERMANOVA *p*-value <0.05. Color legends indicate the type of samples.

Principal coordinate analysis (PCoA) was conducted to investigate the microbial composition differences between and among the various sample groups (Figure 5; Supplementary Figure S3). The analysis revealed distinct community differences between manure samples from the two states, although such differences were not observed in soil and water samples. When combining soil, water, and manure samples from both states into three sample groups, significant differences were observed (PERMANOVA *p*-value 0.0005, Figure 5A). Specifically, significant differences were noted between soil vs. water (*p*-value 0.0016), soil vs. manure (*p*- value 0.0043), and manure vs. water (*p*-value 0.0358) (Figure 5A). No distinct differences were identified between cattle and poultry samples (*p*-value 0.5069, Supplementary Figure S3). However, when samples are grouped into nine different groups by collection states (Alabama and Tennessee), farm type (cattle or poultry), and sample sources (water, soil, and manure), significant group differences were observed (overall *p*-value 0.0034, Figure 5B). The most significant differences were observed between manure samples collected from cattle farms in Tennessee and soil samples collected from cattle farms in Alabama (*p*-value <0.001) (Figure 5B).

### Differential abundance of taxa

**Figure 6:**
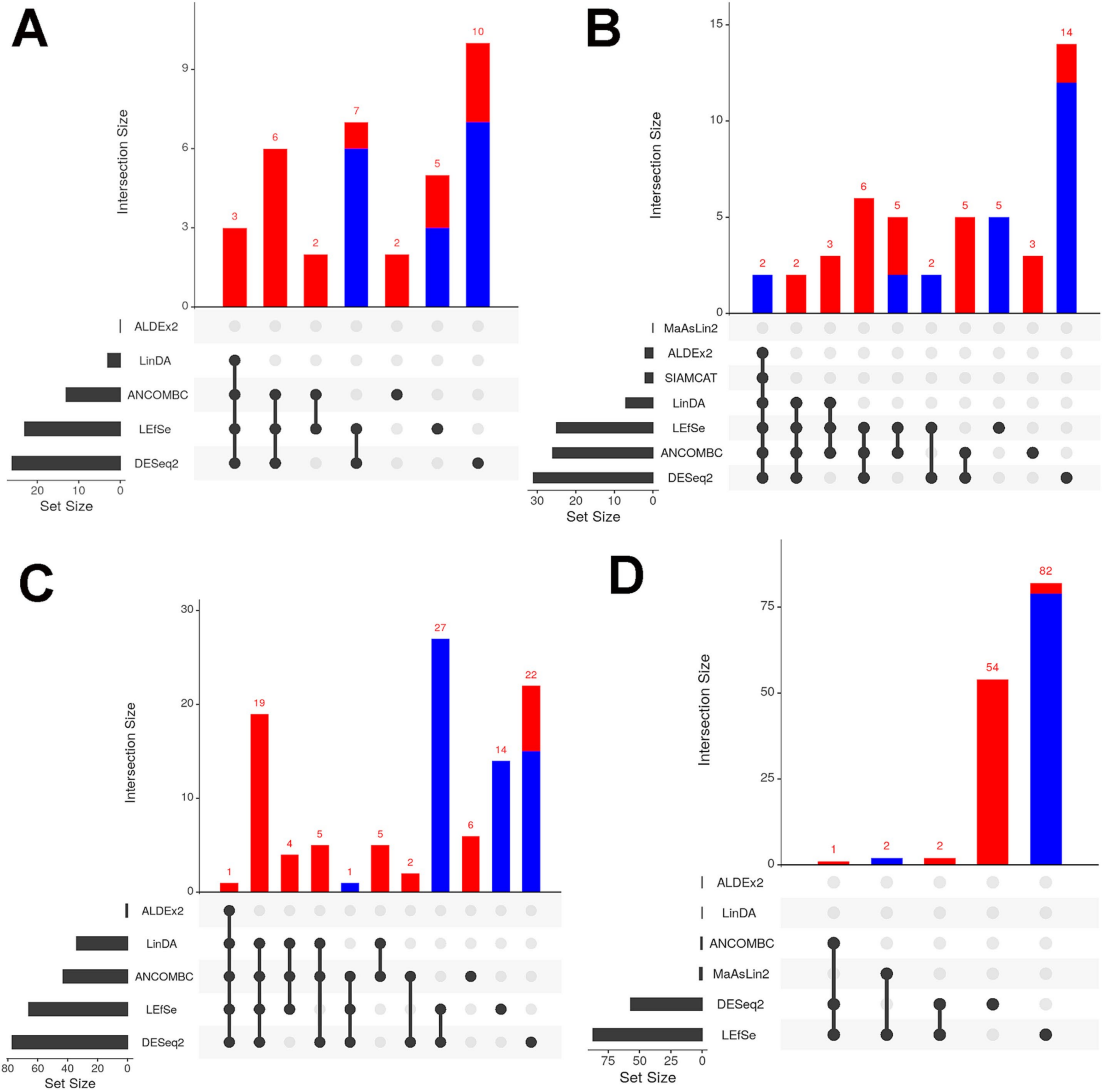
Differential abundance analysis (DAA) of **(A)** water; **(B)** soil and **(C)** manure samples of Alabama and Tennessee. Red bar color represents differential abundance in Alabama and blue color represents differentially abundant in Tennessee. **(D)** DAA between cattle and poultry samples. Red bar color represents differential abundance in cattle farms and blue color represents differentially abundant in poultry farms. Number at the top of each bar indicates the number of differentially abundant genera.

Metagenomic sequencing reveals how microbial populations influence ecosystems, with differential abundance analysis (DAA) identifying key taxa. However, the choice of DAA methods and data preprocessing significantly impact results, necessitating careful handling to ensure accurate interpretations. To address this variability, we employed seven different DAA methods namely DESeq2, LinDA, LEfSe, ANCOM-BC, ALDEx2, MaAsLin2, SIAMCAT, and LinDA. In Alabama, *Deinococcus* and *Ligilactobacillus* were consistently found to be more abundant across all environmental samples, by both DESeq2 and LefSe methods (Figure 6). In water samples, Alabama exhibited higher abundance of *Deinococcus*, *Noviherbaspirillum*, and several other genera including g_WLRQ01 and JZUE_g, *Pseudarthrobacter, Hymenobacter, Spirosoma, Methylorubrum, Sphingomonas* by multiple DAA methods (Figure 6A). Conversely, *Enhydrobacter* and *Caldimonas* were found to be less abundant in Alabama soil samples compared to Tennessee, as reported by six DAA methods, while eleven genera, including *Paracoccus* and *Rubrobacter*, were found to be highly abundant in Alabama by at least three separate DAA methods (Figure 6B). Notably, 28 genera were differentially abundant in Alabama manure samples compared to Tennessee including *Enterococcus* (by five DAA methods, Figure 6C), *Bifidobacterium*, *Georgenia*, *Cellulomonas, Timonella, Oerskovia, Cellulosimicrobium, Isoptericola, Krasilnikoviella, Ornithinimicrobium, Cnuibacter, Glutamicibacter, Dietzia, Rhodococcus, Nocardioides, Thermophilibacter, Kurthia, Lysinibacillus, Jeotgalibaca and Stutzerimonas* (by four methods); *Brevibacterium, Flavimobilis, Microbacterium, Tessaracoccus, Fundicoccus, Lactobacillus, Ligilactobacillus* and *Halopseudomonas* (by three separate DAA methods). In contrast, *Lawsinobacter* were found to be low in abundance in Alabama (by three methods). Among these genera, *Enterococcus* is well known for harboring antimicrobial resistance (AMR) genes including vancomycin. Furthermore, when comparing cattle and poultry samples, *Comamonas* was found to be differentially more abundant in cattle samples compared to poultry samples by three DAA methods (Figure 6D).

### Community virulome

**Figure 7:**
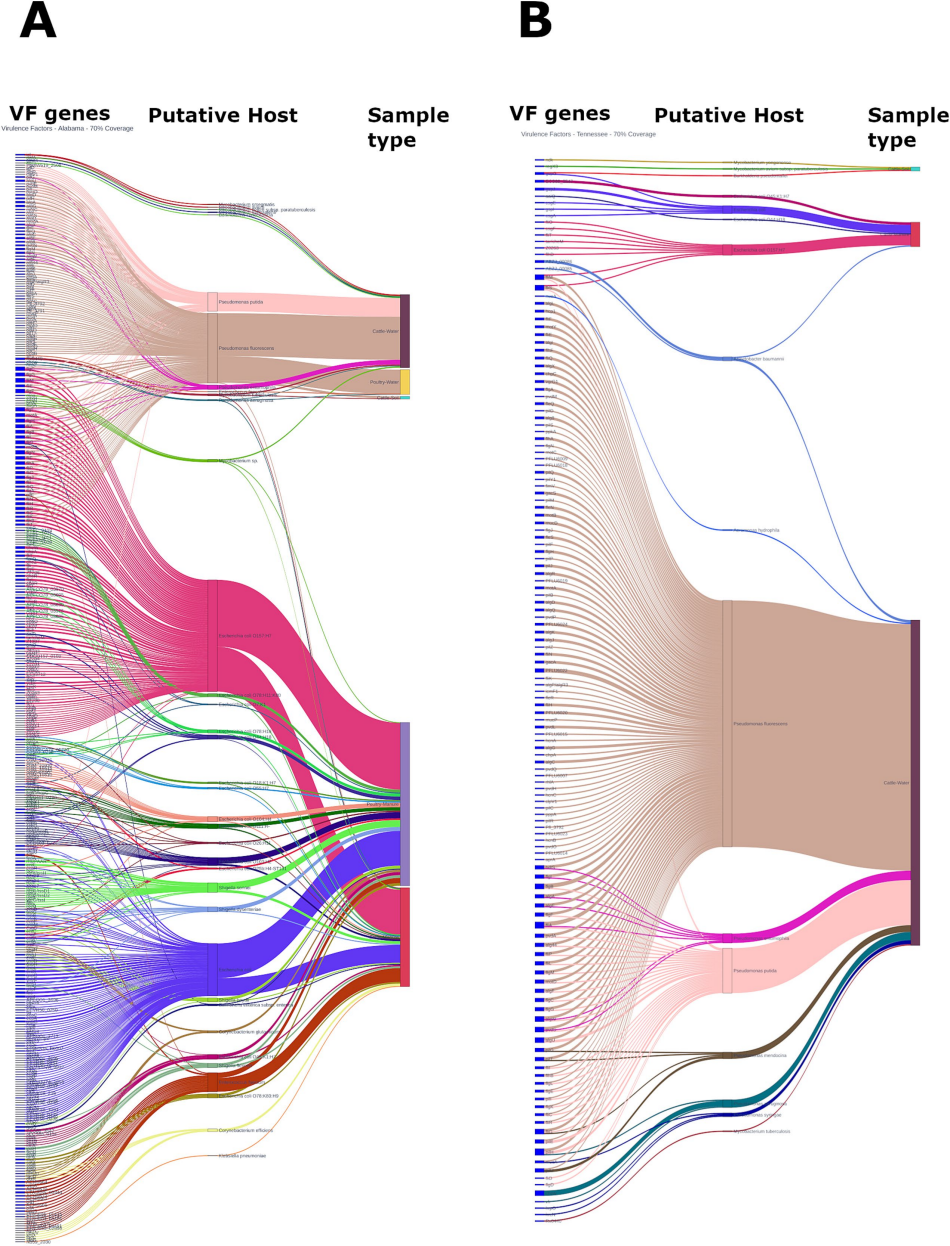
Prevalence of virulence genes and their putative hosts observed in cattle and poultry farming systems in Alabama **(A)** and Tennessee **(B)**. The figure represents VF genes detected using a detection threshold of ≥70% genes coverage.

The community virulome was analyzed using the VFDB reference database, which covers virulence factors (VFs) from 32 genera of common bacterial pathogens. With a 70% gene coverage cut off, our analysis identified 403 distinct VFs across 42 putative hosts species (Figures 7A,B). These VFs encodes 30 major categories of proteins conferring functions such as adhesin, biofilm formation, motility, exotoxin, immune modulation, nutritional/metabolic factor or enzyme, quorum sensing, metal uptake, effector delivery system, and invasion to name a few (Supplementary Figure S4). Remarkably, over 80% of VF genes were detected in Alabama (1,097 out of 1,357), primarily within manure (*n* = 793, from both cattle and poultry farms, 58.44%) and water (from both cattle and poultry farm, *n* = 296, 21.81%) samples. Manure samples harbored a diverse array of VF genes commonly associated with enteropathogenic bacteria. Notably, protein molecules integral parts of various effector delivery systems, including types II, III, IV, V, VI, and VII, were identified in these samples, mostly in poultry manure samples from Alabama, where Gram-negative bacteria are presumed to be the putative hosts. Interestingly, some of the VF genes (e.g., *che*W, Rv0440) identified in manure samples were also detected in soil and water samples. All the VF genes identified, with *E. coli* O157:H7 as the putative host, primarily encode flagellar proteins and are associated with type 3 secretion system, chemotaxis, and biofilm formations. Other frequently detected VF genes in soil and water samples likely originated from *Mycobacterium* spp. including *M. tuberculosis*, and *P. aeruginosa*. Notably, soil and water samples mostly harbored VF genes typically associated with *Mycobacterium* spp. and Pseudomonas spp., along with *Burkholderia pseudomallei*, *B. cepacia* and *Enterococcus faecalis*. Surprisingly, only 0.81% (11 out of the total 1,357) VF genes were detected in soil samples altogether.

The above findings were corroborated by the base abundance analysis of VF genes. In general, total base abundance of VF genes was significantly higher in Alabama compared to Tennessee (88.30% vs. 11.70%, *p* < 0.001, Supplementary Table S5). Among the community members, manure microbiota harbored the highest proportion (54.42%) of VF genes, followed by water (45.44%), and soil (0.14%). Surprisingly, the mean abundance (prevalence as denominator) of VF genes in poultry manure samples was over 5 times higher than the average VF gene abundance across all environmental samples (365,656.383 vs. 1,839,243). Overall, soil samples harbored substantially lower abundance (prevalence as denominator) of VF genes (423 times lower) compared to the overall environmental samples. On the contrary, water samples collected from Alabama exhibited 1.44 and 1.40x higher numbers (prevalence as denominator) compared to all environmental samples and all water samples, respectively.

Microbiota of manure samples from both Alabama and Tennessee were found to harbor VF genes associated with major diarrheagenic pathogens in humans, including *E. coli* O157:H7 and 13 other *E. coli* serovars, also *Shigella sonnei*, *S. boydii*, *S. flexneri*, *S. dysenteriae* etc. (Supplementary Figure S6). Tennessee water samples mostly harbored other VF genes typically found in *Mycobacterium* spp., while VF genes associated with *Pseudomonas* spp. were mostly detected in water samples from both regions, although in lower abundance. Additionally, various other VF genes from putative foodborne pathogens such as *Salmonella enterica*, *Enterococcus* spp., *Burkholderia* spp., and *Aeromonas* spp., as well as human pathogens including *Klebsiella pneumoniae* and *Acinetobacter baumannii*, among others, were detected of discrete samples in both manure and water. In general, the soil samples from both regions exhibited very low abundance of VF genes (Supplementary Figure S5).

### Community resistome

**Figure 8:**
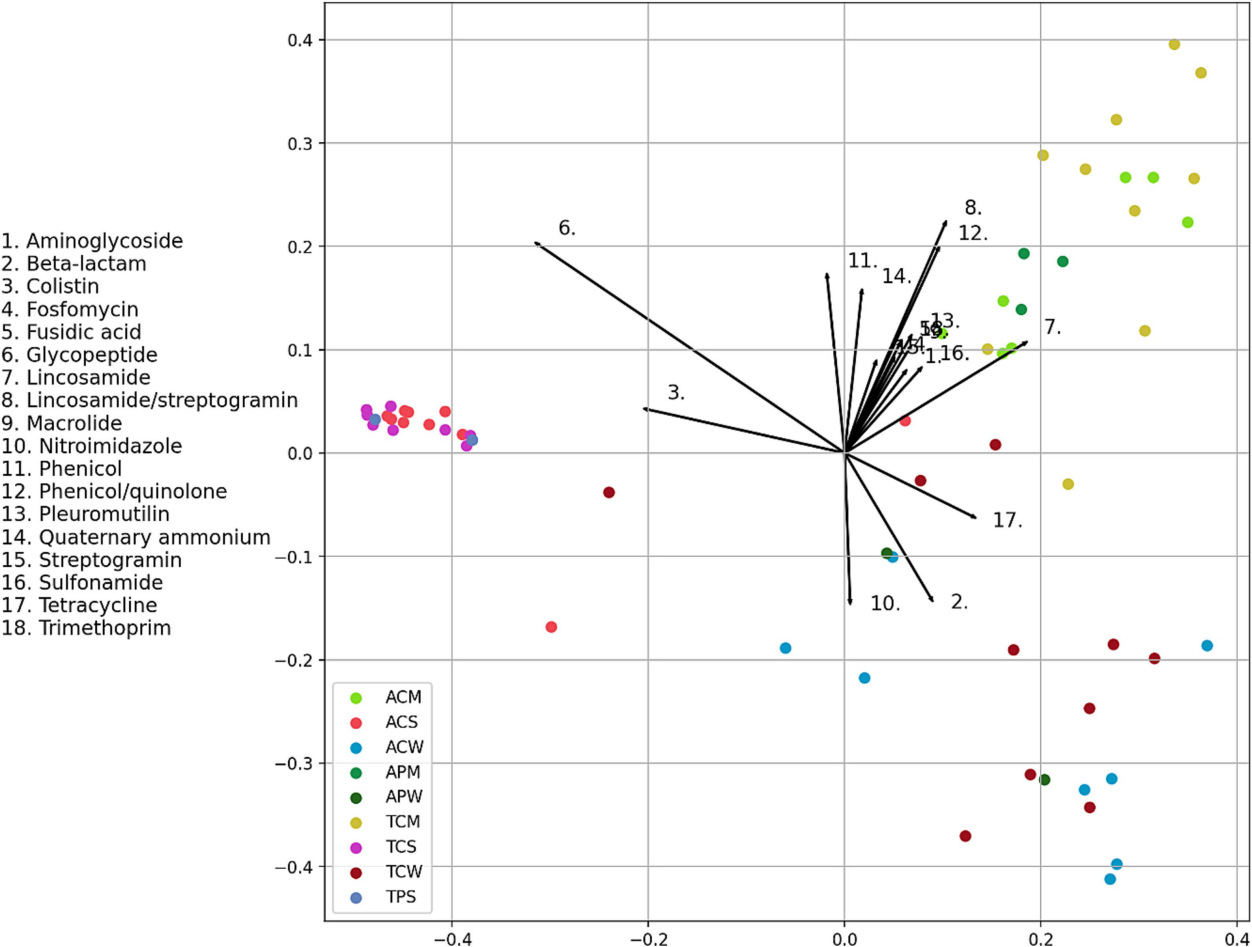
Community resistome profiles observed in soil, manure and water samples collected from cattle and poultry farming systems in Alabama and Tennessee. The figure represents antimicrobial resistance genes (ARGs) detected using a detection threshold of ≥40% genes coverage.

A total of 109 antibiotic resistance (AMR) genes associated with 18 antibiotic class/subclasses were identified using a detection threshold of at least 40% gene coverage (Figure 8). Among those, tetracycline exhibited the highest abundance (58.55%), followed by macrolide (13.99%), streptomycin (4.62%), vancomycin (4.28%), lincosamide (4.23%), cephalosporin (3.51%), beta-lactam (1.81%), carbapenem (1.72%), sulfonamide (1.62%) and pleuromutilin (1.03%) (Supplementary Table S6). Altogether, these 10 classes of antibiotics accounted for over 95% of all AMR gene abundances. In terms of prevalence, vancomycin displayed the highest prevalence, detected in 25 out of 60 environmental samples, followed by tetracycline (21 out of 60), lincosamide (18 out 60), streptomycin (14 out of 60), and macrolide, cephalosporin, carbapenem and sulfonamide (each detected in 12 out of 60 samples). Colistin and tobramycin resistant genes were also observed but with low abundance and prevalence (0.11 and 0.06%, respectively and in 1 out of 60 samples). Notably, colistin is considered as a last-resort antibiotic for treating patients when other drugs are ineffective. Interestingly, tetracyclines, macrolides, streptomycin, lincosamides (specifically pirlimycin), cephalosporins, beta-lactams, and sulfonamides were among the antibiotics used at various times in the history of the participating farms.

The microbial communities in both Alabama and Tennessee exhibited similar abundances of the community resistome (54% vs. 46%, respectively, Supplementary Table S6). All manure samples contained at least one antimicrobial resistance (ARM) gene, while 90% of water samples and in 55% of soil samples harbored at least one AMR gene. Colistin and tobramycin resistance genes were exclusively identified in Tennessee cattle farm water and were absent in Alabama. Quinolone resistance genes were solely detected in Alabama poultry manure microbiota. Sample-wise, manure microbiota harbored over 91% of the AMR genes, in both states, accounting for 50.29 and 41.14% in Alabama and Tennessee, respectively; followed by water (5.25%) and soil microbiota (3.32%). Interestingly, soil microbiota alone harbors three-quarters of the total vancomycin resistance gene abundances shared between the two states (Alabama, and Tennessee, 56.20 and 21.03%, respectively). Surprisingly, soil microbiota in Tennessee harbors no other resistance genes but vancomycin alone (Figure 8).

The average relative abundances of AMR genes were 1.71 times higher in poultry farms compared to cattle farms. However, the uneven collection of samples with 5 poultry samples from Alabama and 2 from Tennessee, compared to 25 cattle samples from Alabama and 28 from Tennessee, due to poultry farm visit restriction during the COVID-19 pandemic, limits our ability to perform a confirmatory statistical analysis.

### Community ecology

#### Envfit analysis

**Figure 9:**
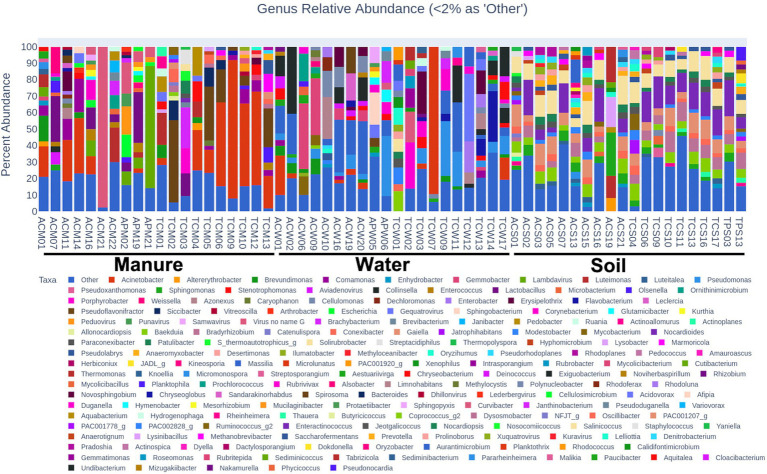
Envfit analysis showing relatedness of environmental samples with antimicrobial resistance (AMR). The length of the arrow indicates the strength of association (significance), while the proximity of samples to the arrows indicates the strength of their relationship. Arrow number indicates the AMR type as mentioned in the legend. Each dot represents individual samples. Color legend indicates the sample type. In each of the five-digit alphanumeric numbers of samples, 1st digit represents the state: “A” for Alabama, “T” for Tennessee; 2nd digit represents farming practices; “C” for Cattle, “P” for Poultry; and third digit represents samples types: “W” for Water; “S” for Soil and “M” for Manure, and finally numeric value represents the individual samples number.

To further assess potential association between antimicrobial resistance and the sample types studied, an envfit analysis were performed which fits sample types onto ordination results derived from multivariate analyses of AMR gene data. The projections of points onto vectors have maximum correlation with corresponding environmental variables, and the factors show the averages of factor levels. Here vectors are antimicrobial resistances and sample types were environmental variables. The length of the arrows indicates the strength of association (significance), while the proximity of samples to the arrows indicates the strength of their relationship (*R*-value). Our analysis revealed that most soil samples (ACS, TCS, and TPS) are clustered together, apart from two samples that exhibited a closer ordination to colistin and glycopeptide (e.g., vancomycin) resistances (Figure 9). Same is true for manure samples. All manure samples (ACM, TCM and APM) clustered in the upper right quadrant, pointing toward several prevalent antimicrobials, including lincosamide, streptogramin, phenicol/quinolone, pleuromutilin, sulfonamide, trimethoprim, aminoglycoside, fosfomycin, fusidic acid and lincosamide. Likewise, all water samples (TCW, ACW and APW) were positioned in the bottom right quadrant pointing toward beta-lactam, nitroimidazole and tetracycline resistances (Figure 9). In general, manure microbiota harbored more diverse AMR genes compared to water and soil microbiota, which could potentially be attributed to observed high diversity of microbial composition in manure samples across both states.

#### Network of ARG subtypes and microbial taxa

**Figure 10:**
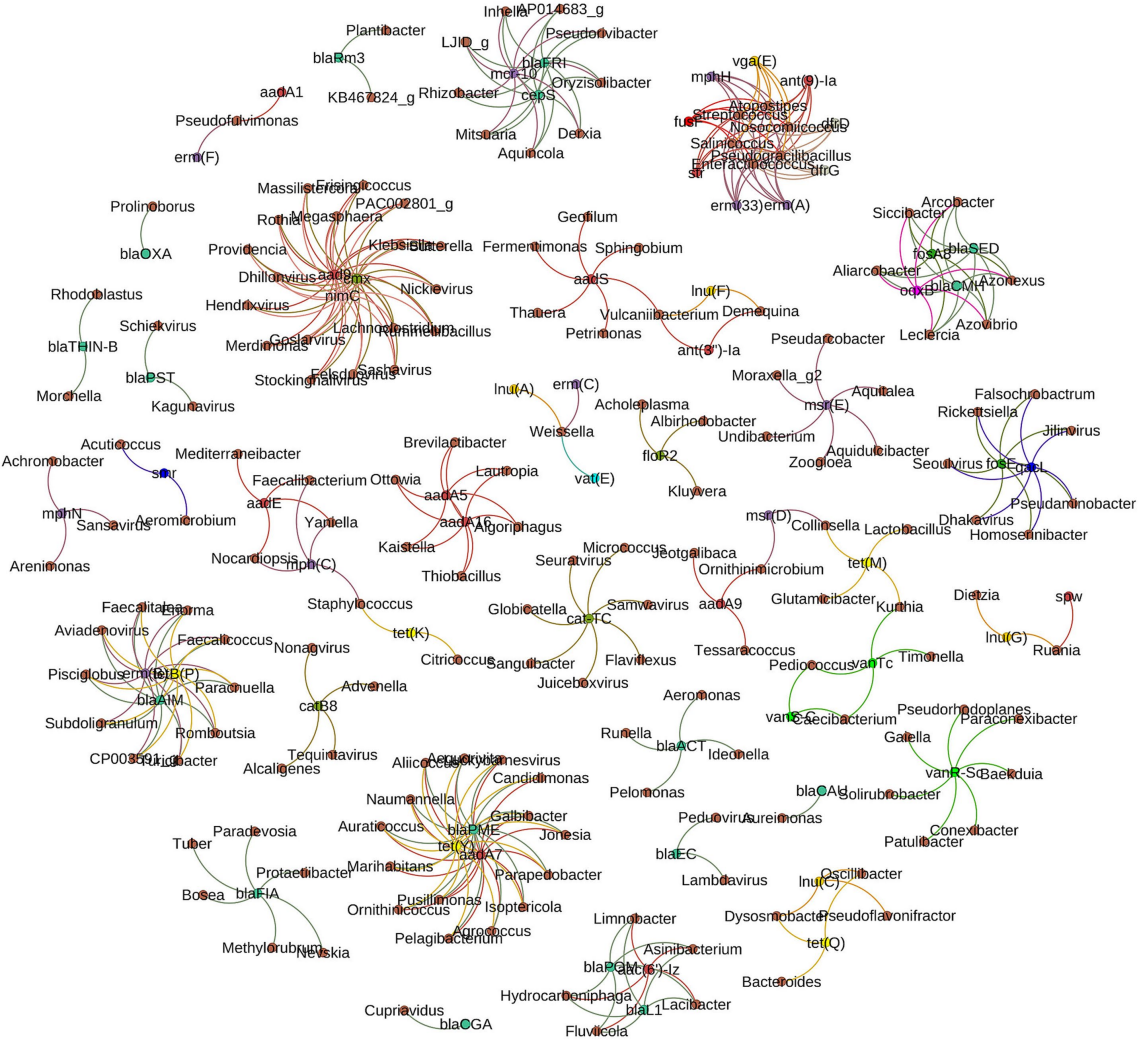
The network analysis revealing the co-occurrence patterns between antimicrobial resistance gene (ARG) subtypes and microbial taxa. The nodes were colored according to ARG types and genus. A connection represents a strong (Spearman’s correlation coefficient *⍴*>0.8) and significant (*p*-value < 0.01) correlation. Antimicrobial resistance genes (ARGs) with 40% or higher coverage are used to generate this figure.

In this study, the hypothesis that patterns of co-occurrence between antimicrobial resistance genes (ARGs) and microbial taxa across different environments might reveal potential host carriers of ARGs were explored. This hypothesis rests on the observation that if ARGs and the co-occurring microbial taxa show significantly similar abundance trends (Spearman’s *ρ* > 0.8, *p*-value <0.01), it suggests specific taxa may harbor these genes, as supported by Forsberg et al. (2014). Our analysis confirmed this hypothesis, with strong correlations and distinct clustering of ARG subtypes and microbial genera forming distinct clusters evident in Figure 10, indicating potential ARG hosts within microbial ecosystems. Notably, the tetracycline resistance gene, *tet*Y, is associated with 15 microbial genera, alongside other ARGs like *bla*PME and *aad*A7 within the same cluster. Remarkably, a bacteriophage, Luckybarnesvirus, is also part of this assemblage, co-occurring with these three ARGs. Additionally, macrolide resistance genes [*erm*(A), *erm*(B), *erm*(C), *erm*(F), *erm*(33), *mph*(N), *mph*(C), *mph*(H), *msr*(D), *msr*(E)] are significantly related to 45 genera across eight clusters, with two bacteriophages included. A distinct cluster shows a combination of nine ARGs, representing resistance to macrolide (*erm*A, *erm*33 and *mph*H), trimethoprim (*dfr*D and *dfr*G), fusidic acid (*fus*F), streptogramin A (*vga*E), streptomycin (*str*) and aminoglycoside (*ant*-9-la), linked to five taxa, including *Streptococcus*. This exemplifies the widespread distribution of resistance genes across various genera, with tetracycline and vancomycin resistance genes notably prevalent, associated with 34 and 11 genera, respectively. Genus *Pseudomonas* has been noted to correlate strongly with multiple ARGs such as *bla*TEM, *qnr*B, and *sul*1 genes, suggesting a nexus of multidrug resistance. Similarly, *Acinetobacter* spp. exhibit connections to acquired ARGs like *arm*A and carbapenem-resistant genes, indicating its role in critical resistance mechanisms. Although both *Pseudomonas* and *Acinetobacter* are known for intrinsic resistance, this study specifically identified acquired resistance genes that contributed to the spread of resistance mechanisms beyond their intrinsic capacities. These acquired ARGs contribute significantly to multidrug resistance. *Escherichia* spp. show correlations with an extended spectrum of *β*-lactamases, represented by genes such as *bla*CTX-M and *bla*SHV. Bacteriophages have emerged within clusters, linked to the dissemination of ARGs such as *mec*A, which is fundamental in methicillin resistance. Genera like *Bacillus* and *Staphylococcus* are visualized in close association with tetracycline resistance genes, denoted by *tet* genes.

Analysis also revealed taxa with multiple ARG associations appearing to be hotspots for a variety of ARGs. For instance, *Klebsiella* is observed to be associated with *bla*KPC, *bla*NDM, and aminoglycoside resistance genes. *Enterococcus* genera are connected to vancomycin resistance genes (*van*A and *van*B) and exhibit links to genes imparting resistance to macrolides. The *bla*NDM gene exhibits associations with several taxa, including *Klebsiella, Escherichia*, and *Acinetobacter*, indicating its prevalence across diverse bacteria. Genes such as *qnr*, conferring quinolone resistance, are linked with a broad spectrum of genera, reflecting the widespread nature of this resistance.

One of the last resort antibiotics for infection treatment is colistin; colistin resistance gene (*mcr*-10) which was found associated with 9 separate genera including *Rhizobacter*, identified from one of ten Tennessee cattle farm collected water. Soil samples were less diverse and harboring lower abundance of antimicrobial resistance genes. However, soil microbiota was rich in vancomycin resistance where three vancomycin resistance genes and gene regulators (*van*R, *van*S and *van*T) were found associated with 11 genera including *Pediococcus*. Among food borne pathogens, *Staphylococcus* was found associated with tetracycline resistance [*tet*(K)]. No other food borne pathogens were captured in this network co-occurrence analysis.

## Discussion

Understanding the dynamics of microbial community, resistome, and virulome across various farm samples such as water, manure, and soil, is important for advancing farming practices and enhancing our understanding of these ecosystems. Previous studies (Durso et al., 2012; Zhu et al., 2013; Forsberg et al., 2014) have emphasized the crucial role of microbial communities in AMR dissemination within agricultural settings and the need for comprehensive surveillance in livestock environments to safeguard public health. Conducting a detailed investigation of microbial diversity, community resistome, and farming practices, this study offers critical scientific insights for a sustainable agricultural system. Demographic trends among cattle and poultry farmers in Tennessee and Alabama, predominantly older males with limited education, impact the adoption of advanced farming practices, including AMR management. Regional differences in manure management and dead animal disposal practices underscore the variations in environmental management strategies between the states. Through thorough analysis, the study provides valuable insights into the interplay of microbial communities within agricultural environments and suggests strategies for mitigating AMR dissemination and optimizing agricultural sustainability.

Multi-kingdom microbiome analysis uncovered over 1950 microbial species across four major kingdoms in Alabama and Tennessee. Bacteria, comprising over 1800 species, emerged as the predominant kingdom, closely followed by viruses, with minimal representations of Archaea and Fungi. High prevalence of bacteria is in concordance with previous study (Pershina et al., 2015) reporting the significant roles of Proteobacteria and Actinobacteria in agricultural soils for nutrient cycling and soil structure maintenance. Interstate variability in bacterial phyla was observed reflecting Proteobacteria being most abundant in both states, with variations in the abundance of other phyla like Cyanobacteria and Actinobacteria. These variations might be a results of distinct environmental and agricultural conditions influencing the soil microbiota (Chen et al., 2018). Notable difference was also observed in the bacteriophage composition across states, highlighting the substantial role of bacteriophages in modulating bacterial populations, influencing nutrient cycling, and overall soil health (Wang et al., 2022; Au et al., 2021; Choudhary et al., 2021; Ye et al., 2019). The high abundance of bacteriophages in cattle manure is concerning due to their established role in AMR transmission (Gummalla et al., 2023; Colavecchio et al., 2017). This could potentially undermine the effectiveness of manure as biofertilizer by altering the microbial communities and promoting the spread of resistance genes, eventually compromising soil health (Ross and Topp, 2015; Roux et al., 2015). Understanding these microbial dynamics is, therefore, crucial for improving agricultural practices and managing microbial ecosystems.

Notable heterogeneity was observed in microbial communities across soil, manure, and water samples, reflecting the significant influence of environmental niches on microbial distribution and function. This findings is consistent with Lopatto et al. (2019), which reported how swine manure application significantly impacted soil microbiome profiles and AMR dynamics. The study further supports the idea that these microbial communities are dynamic entities shaped by geographic and agronomic factors, as also noted by Constancias et al. (2015), who obseved spatial variability in microbial communities related to diverse soil properties and land-use patterns, mirroring the interstate differences observed here. The predominance of Actinobacteria and Proteobacteria in these ecosystems contrasts the predominance of Acidobacteria reported by Nkuekam et al. (2018). This discrete observation may indicate influence of local agricultural practices in shaping the microbiome. The predominance of Actinobacteria in soil samples makes ecological sense due to their known role in organic matter decomposition which contributes to soil fertility and productivity (García-Orenes et al., 2013; Yaradoddi and Kontro, 2021). Similarly, the prevalence of Proteobacteria in aquatic environments aligns with their known ecological adaptability and crucial role in biogeochemical cycles and consistent with van der Heijden and Hartmann’s (2016) finding. However, their known potential as vectors for ARGs raise concerns and emphasize the need for vigilant water quality management (Li et al., 2022a). Additionally, the observed dominance of Cyanobacteria, in Tennessee’s water samples, may indicate nutrient overabundance, particularly nitrogen and phosphorus, and low dissolved oxygen, which could be harmful for aquatic species. The distinct microbial profiles in manure samples from Alabama and Tennessee likely reflects differences in farming practices, such as feed composition and antibiotic use, which influences both microbial populations and ARG profiles (Marutescu et al., 2022). Collectively, these observed heterogeneities in microbial communities among sample types and farming systems underscore the importance of localized management practices tailored to specific microbial ecologies to maintain ecological balance and enhance agricultural safety and productivity.

The exploration of microbial diversity across agricultural ecosystems in Alabama and Tennessee revealed valuable insight into how localized environmental factors and farming practices shape microbial landscapes. Alpha diversity analysis, using Shannon and Faith’s phylogenetic indices, revealed that most Tennessee sample types generally exhibited higher microbial diversity and species richness, compared to Alabama. This difference is likely due to state-specific agricultural practices, environmental policies, or climatic conditions favoring microbial heterogeneity (Fierer et al., 2012). Among the sample types, manure samples consistently showed higher microbial diversity and richness, which is expected as it provides nutrient-rich environments conducive to microbial growth. While such rich microbial ecosystem can enhance nutrient cycling, soil fertility, and resilience against pathogens (Wagg et al., 2014), it also can serve as a reservoir for AMR genes. As a result, high diversity in Tennessee manure samples could be both an opportunity for biofertilization as well as a potential risk for AMR dissemination if not managed properly (Heuer et al., 2011). Lower phylogenetic diversity in water samples, particularly in Tennessee, likely indicate ecological stress or the impact of improper agricultural practices such as antibiotic or chemical use, affecting water quality and runoff management (Acero Triana et al., 2021).

Enterotyping analysis identified four distinct clusters, highlighting the diverse microbial ecosystems within these farm environments. Manure samples, as usual, exhibited the highest variety of enterotypes indicating a rich and complex microbial community that can play a role in shaping soil microbiota (Heuer et al., 2011). In contrast, soil samples displayed fewer clusters, indicating lower microbial diversity, possibly due to the more stable, structured soil environment. This unique ecosystem of farm-associated soils, distinct from natural soil habitats, might be responsible for limiting microbial variations and contributing to the observed lower diversity (Wagg et al., 2014). Water samples demonstrated an intermediate level of diversity, reflecting the dynamic nature of aquatic microbial populations routinely influenced by nutrient availability and water flow dynamics (Wall et al., 2015). State-wise differences in cluster distributions indicate distinct microbial landscapes between Alabama and Tennessee, with Tennessee exhibiting greater microbial diversity, including unique clusters such as E3. The differential microbial landscape observed in this analysis emphasizes the need for targeted management practices to enhance microbial diversity in these ecosystems.

Principal Coordinate Analysis (PCoA) revealed substantial microbial compositional differences among various sample groups, with pronounced differences observed in manure samples from Alabama and Tennessee. This observation aligns with the alpha diversity analysis, which shows relatively lower microbial diversity in Alabama cattle manure compared to Tennessee. Such differences might reflect the variation (more diverse or uniform) of livestock diets being used or other factors like antibiotic or chemical usage and farming practices (Jami et al., 2013; Shawver et al., 2021). Moreover, the significant variation observed among combined soil, water, and manure samples from both states emphasizes the complex dynamics of microbial communities across different environmental compartments within farm ecosystems. These findings highlight the dynamic influence of agricultural practices and environmental factors on microbial diversity, as supported by ecological literature (Fierer et al., 2010), underscoring the importance of studying multiple sample types to achieve a comprehensive understanding of microbial ecology and its implications for ecosystem functioning.

The differential abundance analysis (DAA) unveiled notable variations in microbial taxa across sample types and regions, highlighting their ecological significance. For instance, *Deinococcus* and *Ligilactobacillus* were consistently enriched in Alabama samples, reflecting their known ecological roles. *Deinococcus* is known for its robustness against environmental stressors, contributing to nutrient cycling and ecosystem resilience (Daly et al., 2007), while *Ligilactobacillus* (formerly *Lactobacillus*) spp., plays an important role in food fermentation processes, and thereby influences microbial dynamics in farm ecosystems (Vinderola et al., 2019). The analysis also revealed characteristic microbial signatures in water, soil, and manure samples, illustrating the interplay between microbial communities and environmental factors (Gomez-Alvarez et al., 2009). The presence of *Deinococcus, Noviherbaspirillum, Pseudarthrobacter, Hymenobacter, Spirosoma, Methylorubrum*, and *Sphingomonas* genera in water samples has ecological implications. For instance, *Pseudarthrobacter* and *Hymenobacter* contribute to organic matter decomposition and nutrient cycling in water environments (Gomez-Alvarez et al., 2009), while *Spirosoma* species assist in the breaking down complex organic compounds (Vinderola et al., 2019). *Methylorubrum* and *Sphingomonas* play roles in water quality and environmental remediation by degrading organic pollutants (Fujii et al., 2003), while *Deinococcus* species resist environmental stressors (Daly et al., 2007) and *Noviherbaspirillum* plays roles in nutrient cycling and soil health, all indicative of a healthy aquatic ecosystem (Lin et al., 2013). In soil samples, the differential abundance of *Enhydrobacter* and *Caldimonas* between Alabama and Tennessee has ecological relevance. For instance, *Enhydrobacter* degrades complex organic compounds in soil environments, reflecting variations in organic matter availability or soil nutrient composition between the regions (Premalatha et al., 2015). Conversely, the higher abundance of *Paracoccus* and *Rubrobacter* in Alabama soils may indicate potential ecological adaptations to local conditions. *Paracoccus* contributes to soil fertility by enhancing nitrogen and phosphorus availability (Zeng et al., 2022), while *Rubrobacter* plays a crucial role in soil organic matter decomposition and nutrient cycling (Raimi et al., 2023). In manure samples, the differential abundance of *Enterococcus, Bifidobacterium*, and *Lactobacillus* suggests implications for agricultural ecosystems and human health (Arias and Murray, 2012; Turroni et al., 2018; Afanador-Barajas et al., 2021). *Enterococcus* indicates potential risks of AMR gene transfer to environmental bacteria (Arias and Murray, 2012), *Bifidobacterium*, known for its probiotic properties, suggests fecal contamination (Turroni et al., 2018), *Lactobacillus* spp. influence microbial community structure and nutrient cycling in agricultural soils (Afanador-Barajas et al., 2021), while *Rhodococcus* and *Nocardioides* contribute to organic matter decomposition and nutrient cycling in manure-amended soils (Ma et al., 2021). Moreover, the differential abundance of *Comamonas* between cattle and poultry samples underscores farming practices’ influence on microbial community dynamics, with *Comamonas* species commonly inhabiting animal intestinal microbiomes (Ryan et al., 2022).

The identification of at least 82 distinct virulence factor (VF) genes, encompassing diverse functional categories such as adhesion, biofilm formation, motility, toxin production, immune modulation, nutritional/metabolic factors, effector delivery systems etc., in this study underscores the dynamic nature of the community virulome and reflect the multifaceted strategies employed by microbes to colonize hosts and cause disease (Wu et al., 2008). Notably, 70% of these genes were detected in Alabama, primarily in manure and water samples from cattle and poultry farms, reflecting the impact of agricultural activities on the environmental virulome (Li et al., 2022b). The detection of VF genes related to effector delivery systems in poultry manure samples from Alabama suggest potential spread of VFs within the microbial community (Curtis et al., 2005). The overlap of VF genes in soil and water samples with those in manure points to contamination through runoff, indicating potential VF dissemination beyond agricultural areas (Wu et al., 2008). Interestingly, the various non-pathogenic VF genes, typically associated of *E. coli* O157:H7, *Shigella* spp., and *Salmonella* spp., were detected, but their major pathogenicity factors like intimin (*eae*) and shiga toxin (*stx*) remined undetected. This suggests that these non-pathogenic trait-associated VFs are perhaps widespread within environmental microbial communities, serving basic survival and fitness functions across diverse bacterial communities (Reiland et al., 2014). Detection of *Salmonella enterica rpo*S gene, essential for its survival under stress, indicates that infection determinants may also confer benefits for bacterial survival outside the host (Abdullah et al., 2017). Additionally, VF genes from *Mycobacterium, Pseudomonas, Burkholderia*, and *Enterococcus* in soil and water samples highlights the vast genetic diversity in these ecosystems (King et al., 2017; Egberongbe et al., 2023; Padilla and Lobos, 2013). The detection of VF genes associated with diarrheagenic pathogens in manure samples as well as putative foodborne pathogens in water samples highlights public health risks, emphasizing the need for rigorous water quality monitoring and pretreatment before agricultural use to minimize transmission risks and ensure environmental health and safety (Rodrigues et al., 2020; Sen and Rodgers, 2004). Finaly, it is important to note that mere presence of these VF genes does not necessarily indicate the presence of pathogens but may indicate the potential carriage of these genes by various community members. This highlights the vast reservoir of genetic diversity within natural microbial populations, providing opportunities for ceratin pathogens to acquire, assemble, and exploit a subset of these genes for survival, fitness, or pathogenicity within their ecological niche or when encountering a suitable host (Baquero et al., 2022; Mattock and Blocker, 2017).

The community resistome analysis identified 109 ARGs associated with 18 antibiotic classes/subclasses, with tetracycline being the most abundant. Its high abundance is consistent with its widespread usage in human and veterinary medicine, as well as its persistence in the environment (Martinez, 2009; Bengtsson-Palme et al., 2017). Vancomycin emerged as the most prevalent resistance gene, raising concerns about its potential transfer to clinically relevant pathogens, given the interconnected nature of human, animal, and environmental health. This emphasizes the need for robust surveillance and mitigation efforts (Wegener, 2012). Soil samples exhibited predominant resistance to colistin and vancomycin, reflecting environmental antibiotic resistance trends (Bengtsson-Palme et al., 2017), while manure sample exhibited resistance to a wide range of antibiotics, highlighting the impact of agricultural practices on resistance dynamics (Durso et al., 2012; Holmes et al., 2016). Water samples primarily harbored genes associated with beta-lactam, nitroimidazole, and tetracycline resistance, suggesting potential dissemination through water sources (Larsson, et al., 2018). The exclusive detection of colistin and tobramycin resistance genes in Tennessee cattle farm water, despite their rare use in cattle farming, suggest potential contamination from hospitals wastewater or improper pharmaceutical disposal (Olaitan et al., 2014). Conversely, quinolone resistance genes solely in Alabama poultry manure suggests specific management practices or selective pressures driving dissemination of these genes (Wang et al., 2017). Overall, high prevalence of ARGs in farming systems raises concern for ecosystem health and food safety, posing risks of multidrug-resistant pathogens emergence and contamination of food products with antibiotic-resistant bacteria, threatening public health (Larsson, et al., 2018; Holmes et al., 2016; Bengtsson-Palme et al., 2017).

The cohesive clustering of soil samples around colistin and glycopeptide (e.g., vancomycin) resistance genes in the envfit analysis suggest potential contamination from anthropogenic sources, as these genes are uncommon in soil under natural conditions (Li et al., 2009; Zhu et al., 2017; Zhu et al., 2013). The clustering of manure samples toward a diverse array of antimicrobials correlates with high microbial diversity and species richness observed in diversity analysis and aligns with prior research (Martinez, 2009; Durso et al., 2012). Such prevalence of diverse antimicrobials likely arises from extensive antimicrobial use in livestock farming, leading to the accumulation of antibiotic residues and active drug compounds in animal waste, which subsequently contaminates manure (Durso et al., 2012). These findings underscore the need for caution when using manure as fertilizer. The clustering of water samples around beta-lactam, nitroimidazole, and tetracycline resistances genes suggests that farm aquatic environments may serve as reservoirs for these resistance genes. The occurrence of beta-lactam and tetracycline resistance likely reflects their extensive use in livestock farming (Bengtsson-Palme et al., 2017; McEwen and Fedorka-Cray, 2002). In contrast, the presence of nitroimidazole resistance, because of its uncommon use in agriculture, may indicate their environmental presence and contamination from wastewater (Löfmark et al., 2005). Further research is needed to evaluate their prevalence in Alabama and Tennessee water sources (Malayil et al., 2022; Bergeron et al., 2017).

This study specifically focused on identifying acquired resistance genes, due to their potential for horizontal gene transfer and contribution to multidrug resistance. The comprehensive network analysis of AMR genes and microbial taxa revealed widespread distribution of ARGs across diverse microbial taxa, defing the notion of single-source ARG origins. Genes associated with tetracycline and vancomycin resistance showed associations with broad range of genera (34 and 11 genera, respectively). This pattern demonstrates extensive dissemination of acquired ARGs within microbial communities through horizontal gene transfer. The association of ARGs such as *bla*KPC, *bla*NDM, and quinolone resistance genes with multiple genera including *Pseudomonas, Acinetobacter*, and *Escherichia*, corroborates findings from clinical isolates (Cantón et al., 2012) and suggests a convergence of resistance patterns across clinical and environmental realms. This is particularly concerning for notorious genera like *Klebsiella* and *Enterococcus* due to their roles in nosocomial infections and as hubs for ARGs (Mathur and Singh, 2013). The co-occurrence of ARGs with heavy metal resistance and biocides, as also seen in both clinical and environmental microbes (Pal et al., 2016), points to an interconnected resistance network that poses public health risks. The co-occurrence of ARGs like *aad*9, *nim*C, and *cmx* with multiple bacterial genera and bacteriophages, including those infecting *Escherichia* and *Salmonella*, illustrates the role of phages in ARG dissemination (Gummalla et al., 2023). The association of resistance genes *mcr*-10, *bla*FRI, and *cep*S with seven bacterial genera, and the co-occurrence of *fos*A8, *bla*SED, *oqx*B, and *bla*CMY in five other genera, further illustrate the intricate resistance networks within microbial communities. The notable presence of *tet*Y, *bla*PME, and *aad*A7 across 14 bacterial genera and the bacteriophage *Luckybarnesvirus* as well as the co-occurrence of genes like *erm*B, *tet*B(P), *bla*AIM, *bla*POM, *aac*(6′)-Iz, and *bla*L1 suggests a complex, multifaceted resistance landscape. The unique resistome of soil microbiota, particularly rich in vancomycin resistance, is consistent with previous findings that suggest soil as a potential reservoir for ARGs (Aminov et al., 2001). The linkage of these genes to genera like *Pediococcus*, known for its role in food fermentation, raises questions about the potential transmission routes of ARGs into the human microbiome (Clewell et al., 2024). Similarly, the association of *Staphylococcus* with the tetracycline resistance gene *tet*(K) underscores the possible ARG transmission via the food chain (Smith et al., 2002). Finally, our findings advocate for a comprehensive ‘One Health’ approach, emphasizing the need to monitor environmental, foodborne, and phage-mediated ARG transmission for effective AMR mitigation strategies.

In conclusion, this study provided deep insights into the complex dynamics of microbial communities and resistome, influenced by geographical, agricultural, and livestock management factors. The findings reveal substantial microbial diversity, demonstrating the intricate nature of these ecosystems and the necessity for specialized management strategies to enhance soil health, agricultural productivity, and environmental sustainability. The detection of diverse virulence and AMR genes, along with bacteriophages in manure, underscores the need for continued surveillance and further research into their genetic roles and transmission dynamics. The presence of cyanobacteria in Tennessee water additionally stresses the importance of continuous water quality monitoring, especially concerning the risk associated with cyanotoxins. The study also emphasizes the importance of targeted education to improve AMR management among farmers. However, limitations exist, such as cross-sectional design and uneven sample distribution between cattle and poultry, which may hinder comparability, causal inferences, and generalizability of findings. Longitudinal studies with balance sample distribution could offer more insights. While the study reported a broad array of ARGs, VFs, and bacteriophages, and their potential association with diverse microbial taxa, it did not assess their functional potential or transfer mechanisms. Future research should study dissemination and expression dynamics of ARGs and VFs. Moreover, the significant presence of bacteriophages prompts further study into other viruses, especially enteroviruses, often overlooked in farm settings. Finally, the findings from this metagenomic investigation should be considered as predictive biology, thus experimental validation is necessary to clarify the roles of specific ARG, VF, bacteriophages, and microbes. Overall, this research advocates for ongoing microbial surveillance and proactive interventions to ensure the long-term sustainability and safety of agricultural systems.

## Data Availability

The raw metagenomic sequencing data generated in this study have been deposited in the NCBI under BioProject number PRJNA1113660 (https://www.ncbi.nlm.nih.gov/sra/?term=PRJNA1113660). The bioinformatics scripts used in this study were made available through a GitHub repository (https://github.com/EzBiome/CMMCRSFS).
