# Supplementary material for: Comparative metagenomics of microbial communities and resistome in southern farming systems: implications for antimicrobial stewardship and public health
Source: Front Microbiol. 2024 Nov 26;15:1443292. doi: 10.3389/fmicb.2024.1443292 (PMC11628260; doi:10.3389/fmicb.2024.1443292)
Supplement: Supplementary file 2 [file Data_Sheet_1.docx]

1. **Supplementary Data**

**Observed Management Practices in Cattle and Poultry Farms in Tennessee and Alabama:**

A total of 26 questionnaires and 29 questionnaires were administered to small and medium-sized cattle and poultry farmers in Alabama and Tennessee respectively. As illustrated in below tables, in the demographics section, majority of the farmers in both States were male (TN= 79.3%, AL= 92.3%) and had education level up to high school diploma (TN = 69%, AL= 42.3%). Also, it was observed that most of the farmers in Tennessee (37.9%) and Alabama (57.7%) were of age 65 and above. On the other hand, it was reported that the percentages of female farmers (TN= 20.7%, AL= 7.7%), university graduates (TN=13.8%, AL= 15.4%) younger cattle and poultry farmers (35 and under) in TN= 3.4%, AL= 3.8% was moderately low. In this survey, it was observed that cattle and poultry farmers use antibiotics mostly for therapeutic and prophylactic purposes (treat sick animals and prevent diseases - 82.4%) in TN, while in AL (treat sick animals only- 69.2%, treat sick animals and disease prevention- 23.2%). Our results were consistent with those reported by Joshua et al., (2018) that antibiotics were mostly administered to prevent and treat diseases in a flock at 63.3%. In Tennessee, 82.8% of the farmers indicated that they maintain written antibiotics use records while in Alabama 57.7% of the farmers did. Results from our study were higher than those recorded in a study by Sawant et al., (2005) where only 50% of the farmers maintained written records on their farms. In both States, an important observation that most of the producers consult veterinarians on antibiotics use on their farms was made, with 86.2% in Tennessee and 76.9% in Alabama. Similar findings showing that 88% of the producers sought veterinarians or other experts' advice before administering antibiotics were reported by VE & C, 2016. This is very encouraging because previous studies have highlighted the involvement of veterinarian's services as fundamental in the global mitigation of antimicrobial resistance in cattle and poultry farms (Hedman et al., 2020). In this survey, all the cattle and poultry farmers in Alabama indicated that they use antibiotics as recommended (100%), however, in Tennessee only 72.4% of the farmers used antibiotics as recommended, 17.2% administered multiple doses, and 3.4% only once or repeatedly until the animal is cured. Contrary to our results, in a study by Ozturk et al., (2019), 59% of the farmers administered antibiotics until the animal was cured, while 45% continued treatment with higher or more regular dosage until the animal displayed signs of cure. our surveys also revealed that most of the cattle and poultry farmers are not fully aware of the implications that result due to antimicrobial resistance. This was exhibited through the responses given to the question of antimicrobial resistance being a public health threat. In Tennessee and Alabama (65.4%, 69%) respectively answered yes while the rest said no. Therefore, there’s a need to educate the small and medium cattle and poultry farmers on the usage of antibiotics on their farms and the implications which arise because of antimicrobial resistance. Our results were slightly lower than those reported by Ozturk et al., (2019), where 77% of the producers were aware that antimicrobial resistance is an important public health concern. It was also recorded that 84.6% in Alabama and 82.8% in Tennessee of the producers in Alabama had been trained on Best Management Practices. In Alabama, 100% of the respondents consulted with extension agents, with 34.6% consulting regularly, 7.7% occasionally, and 3.8% monthly, yearly, only when needed, and often as recommended. However, in Tennessee only 72.4% consulted with extension agents, with majority doing it monthly (31%), often as recommended (24.1.%), regularly (13.8%), only when needed, never, monthly, and yearly at (3.4%), 20.7% of the farmers did not respond to this question. All the cattle and poultry farmers in Alabama disposed their dead animals through the dead animal service. These findings were different from Tennessee where 51.7% was done through dead animal service, 27.6% practiced deep burial, 17.2% above-ground, and 3.4% composting. It was observed that the numbers of the farmers in both States who had fresh produce grown next to livestock was relatively low at 10.3% in Tennessee and 15.4% in Alabama. This is a good agricultural practice that helps to mitigate the dissemination of antimicrobial resistance bacteria from animals to humans through the consumption of fresh produce grown next to the farms. Results from farms in Alabama, indicated that all the farmers practiced stockpiling as a way of manure management. However, in Tennessee various methods were practiced including stockpiling (72.4%), composting (20.7%), collect and spray on hay fields (3.4%) while 3.4% did not practice any manure management practices. Manure has been identified as one of the major ways of transferring antimicrobial resistance bacteria to the environment and humans. Proper manure management such as stockpiling and composting are some of the ways of degrading antibiotic residues in manure(Oliver et al., 2020). From the surveys, a correlation between veterinarian’s advice sought on antibiotics use, and antibiotics used in the farm was observed. This emphasizes the importance of involving veterinarians in the cattle and poultry to provide guidance on the application of antibiotics in the farms.

1. **Supplementary Figures and Tables**

**2.1 Supplementary Figures:**


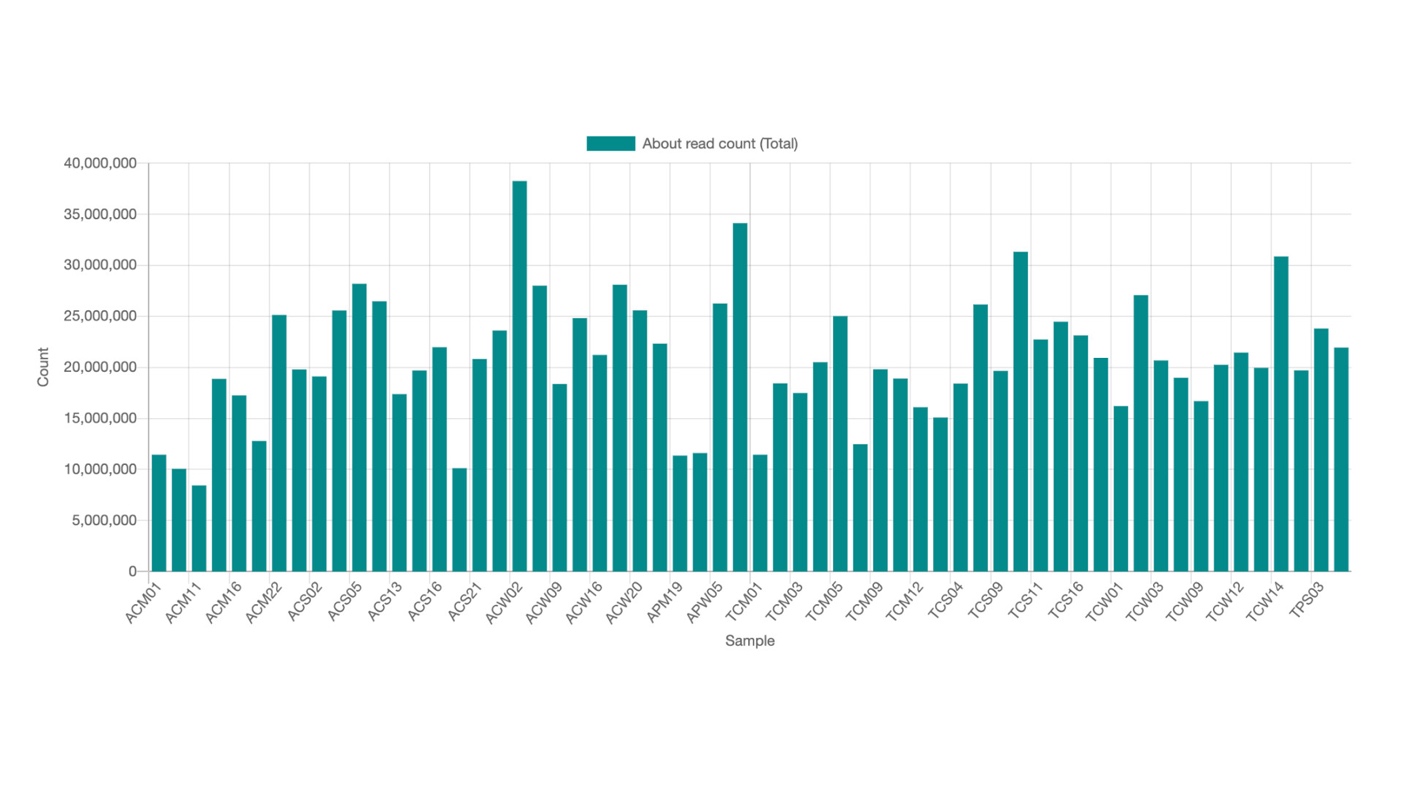


**Supplementary Figure 1:** Read statistics of metagenomic sequenced environmental samples from Alabama and Tennessee cattle and poultry farming systems (n=60). In each of the five-digit alphanumeric numbers of samples, 1st digit represents the state: “A” for Alabama, “T” for Tennessee; 2nd digit represents farming practices; “C” for Cattle, “P” for Poultry; and third digit represents sample types: “W” for Water; “S” for Soil and “M” for Manure, and finally numeric value represents the individual samples number.


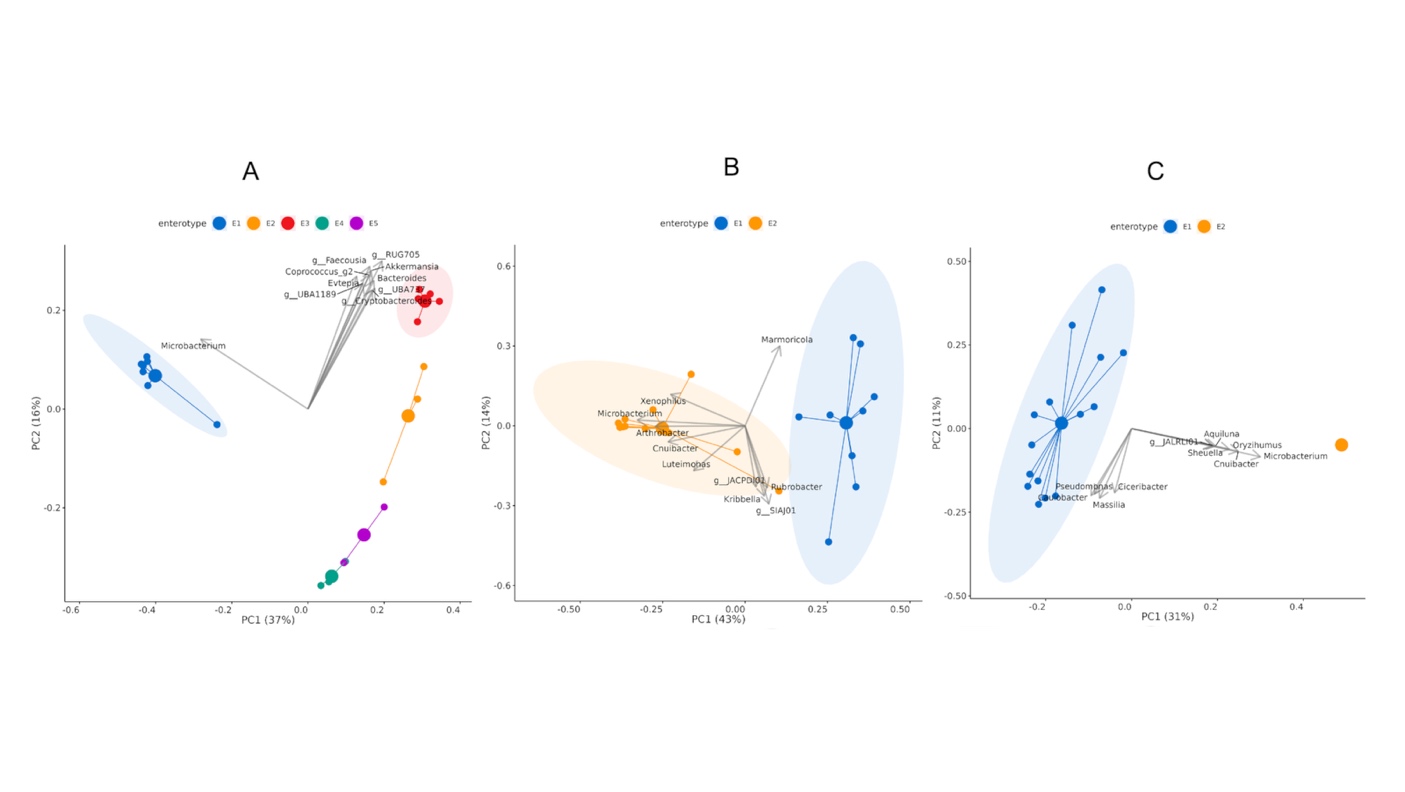


**Supplementary Figure 2:** PAM clustering of enterotypes of environmental samples in genus level collected from cattle and poultry farms in Alabama and Tennessee. A) Manure; B) Soil and C) Water. First two principal coordinates were shown.


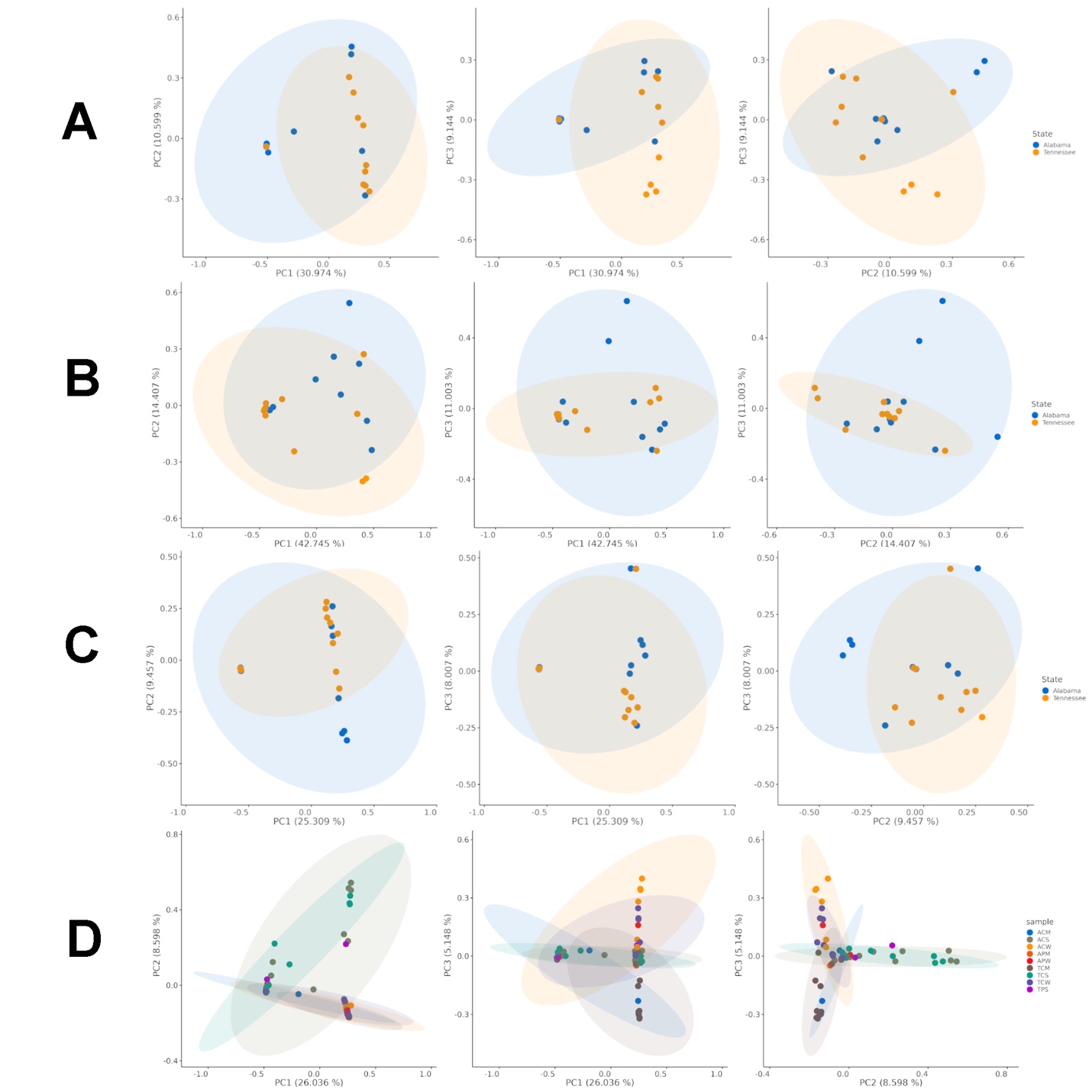


**Supplementary Figure 3:** Beta diversity of soil, water and manure samples across states and farm types. Figure 3A, 3B and 3C represent the first three principal coordinates of manure, soil, and water samples, respectively. Figure 3D represents the same in cattle and poultry samples.


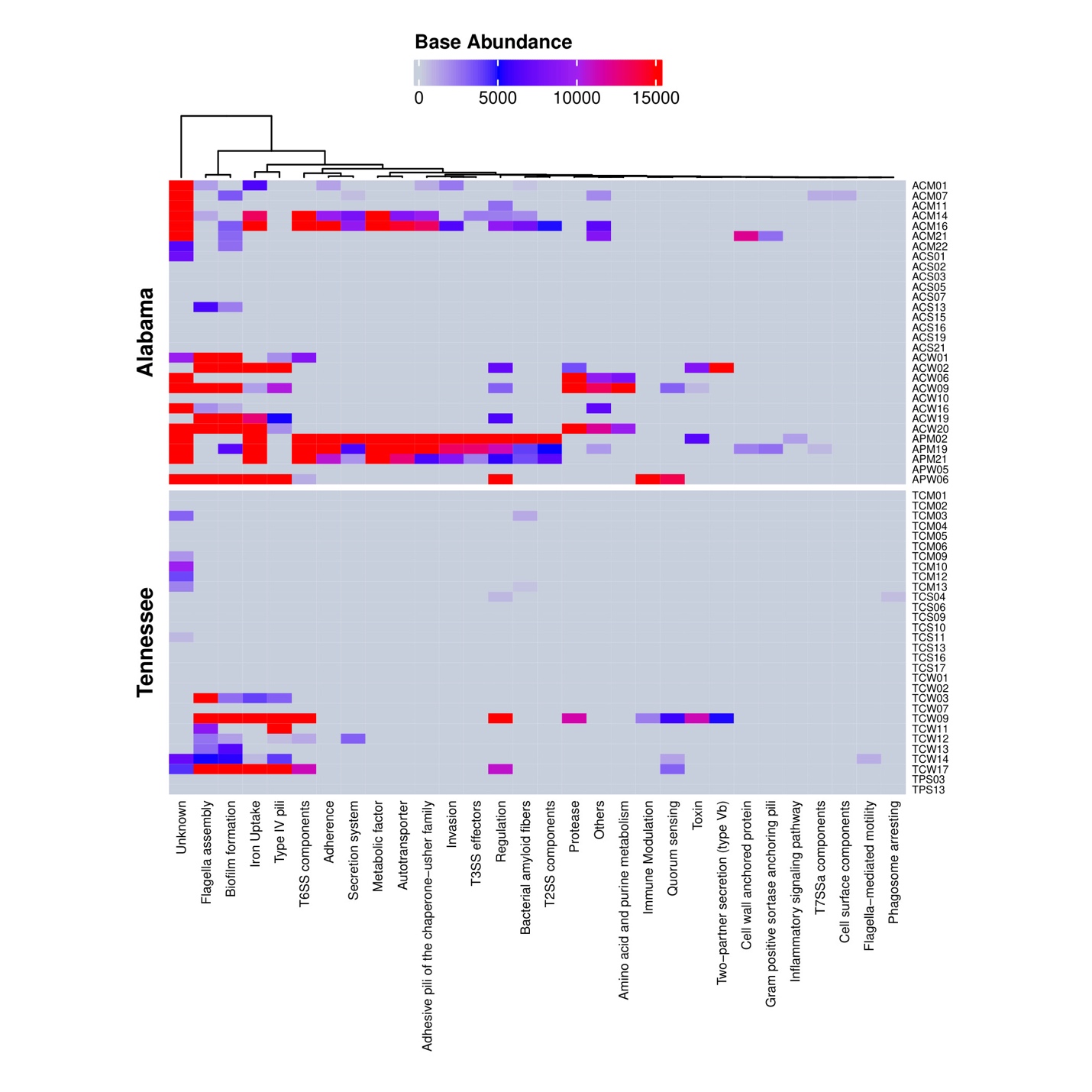


**Supplementary Figure 4:** Base abundance of major virulence factors/gene(s) in individual manure, soil and water sample in Alabama and Tennessee. Virulence genes having 70% or higher coverage are used to generate this figure.


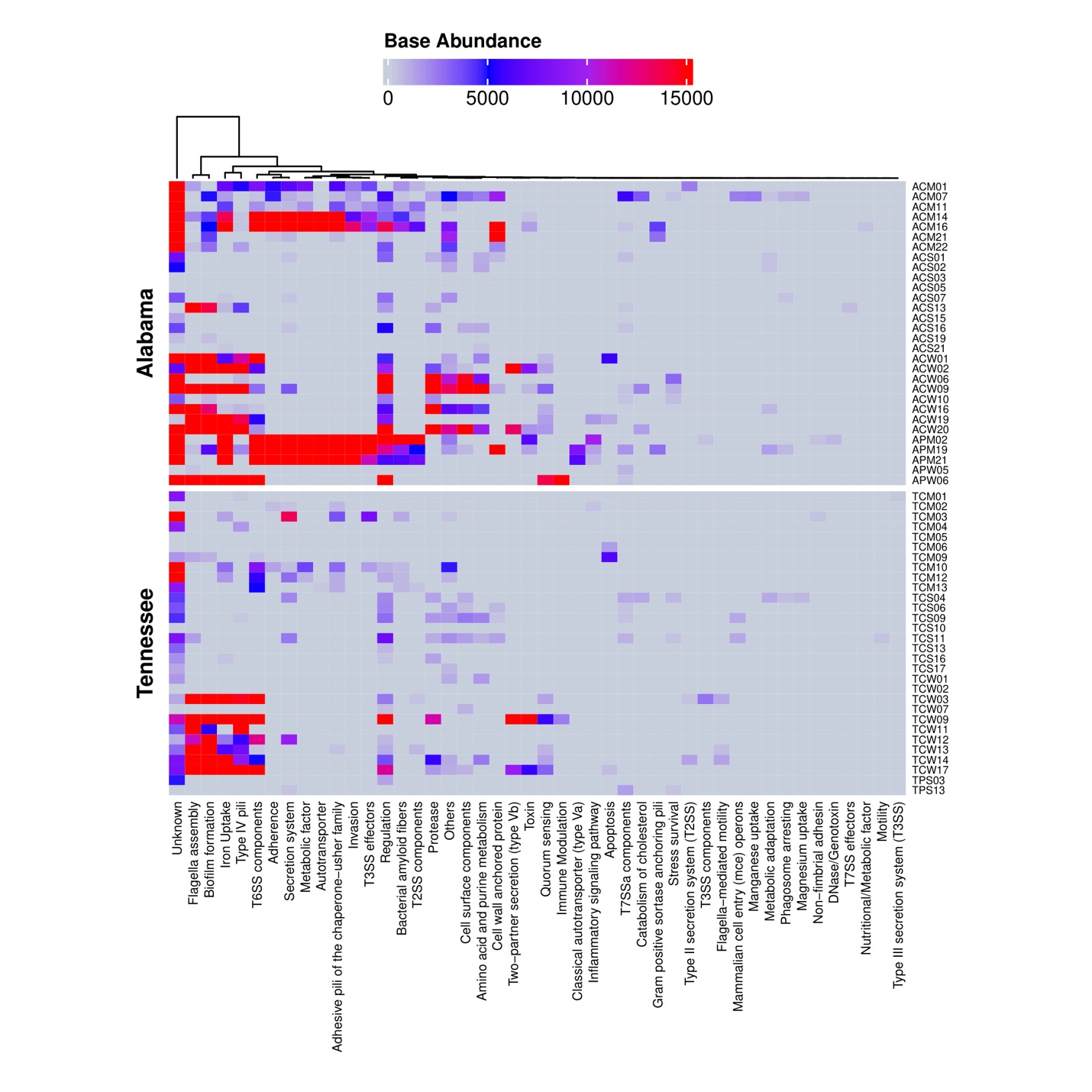


**Supplementary Figure 5:** Base abundance of virulence factors/gene(s) in individual manure, soil and water samples in Alabama and Tennessee. Virulence genes having 40% or higher coverage are used to generate this figure.


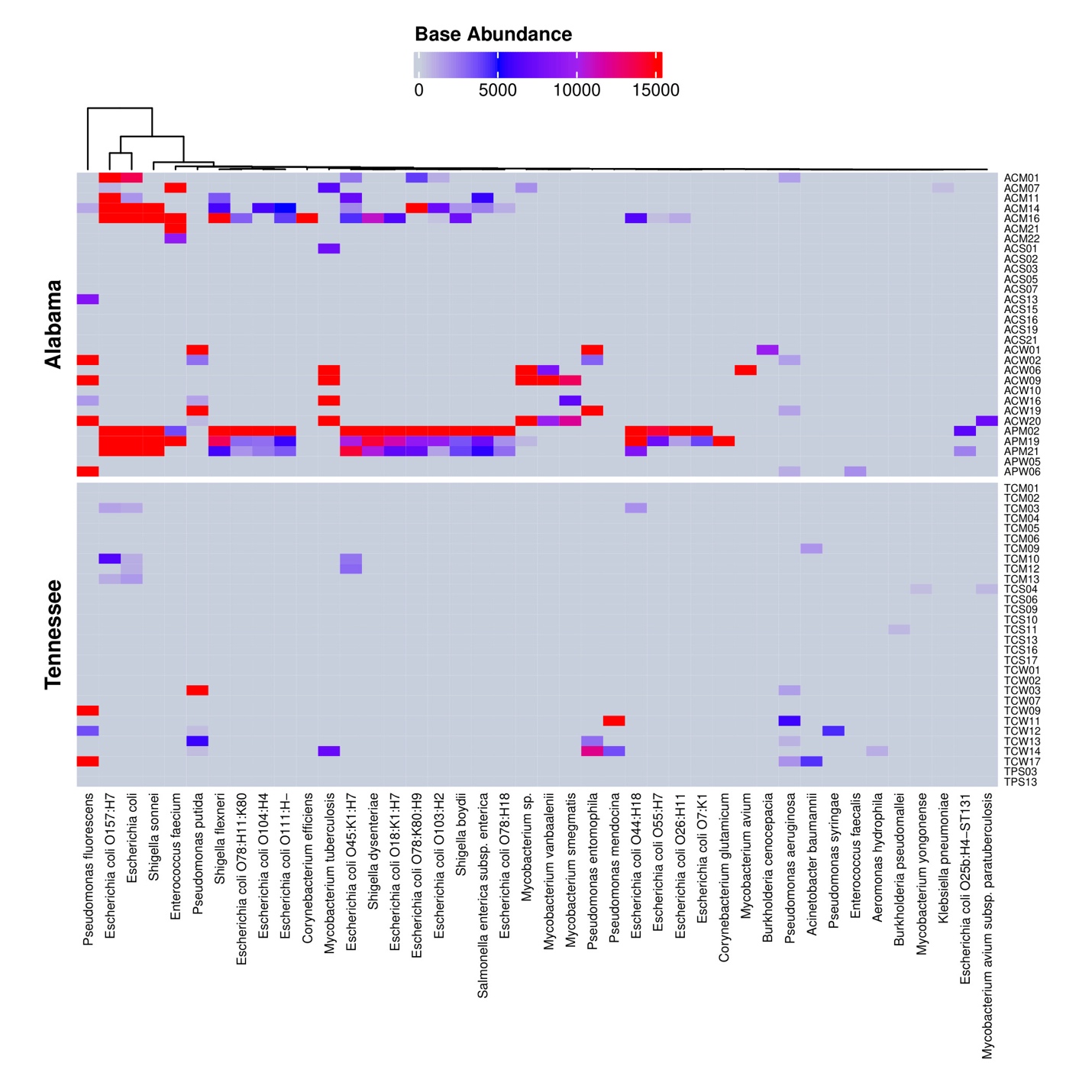


**Supplementary Figure 6:** Putative host based base abundance of virulence gene(s) in individual manure, soil and water samples in Alabama and Tennessee. Virulence genes having 70% or higher coverage are used to generate this figure.


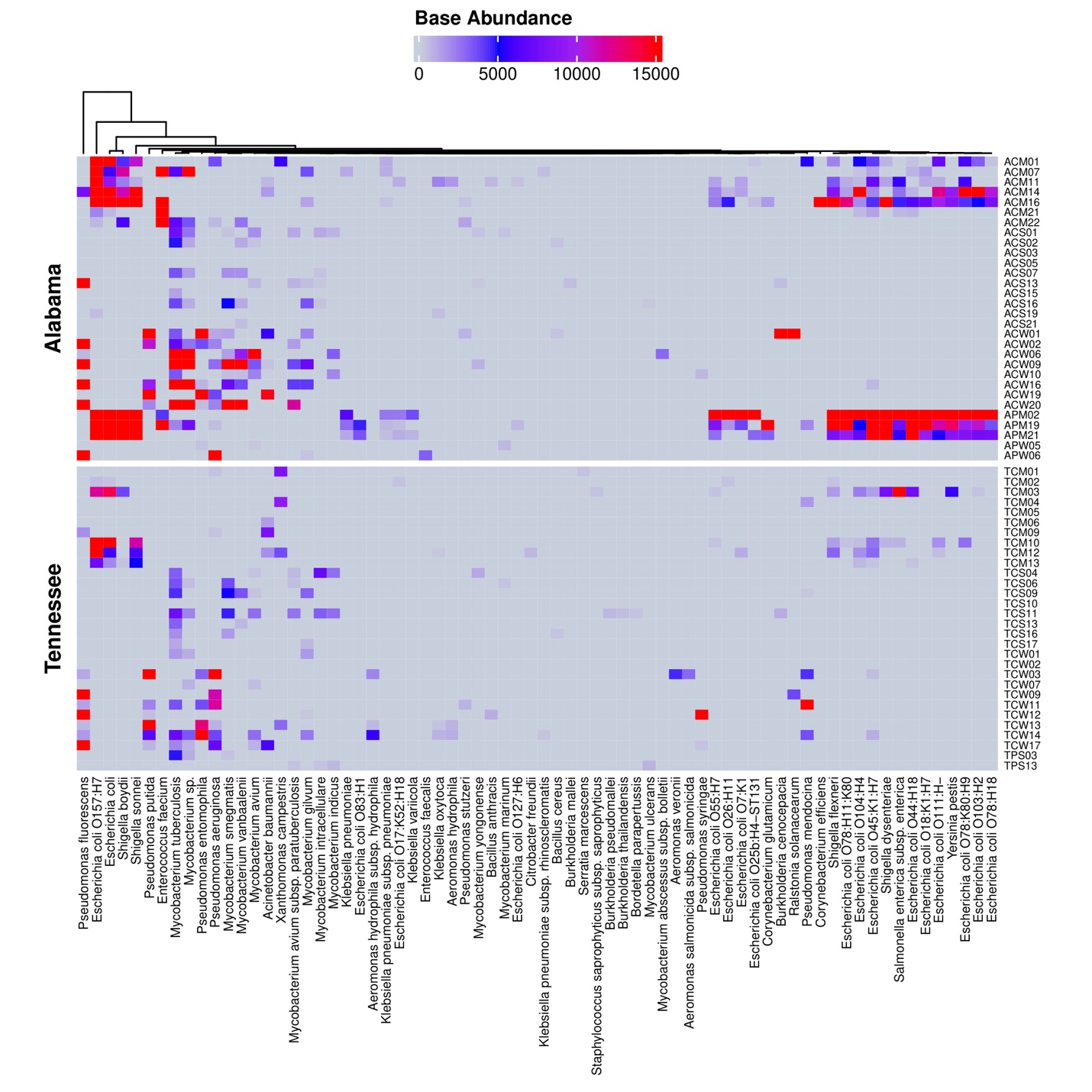


**Supplementary Figure 7:** Putative host based base abundance of virulence gene(s) in individual manure, soil and water samples in Alabama and Tennessee. Virulence genes having 40% or higher coverage are used to generate this figure.


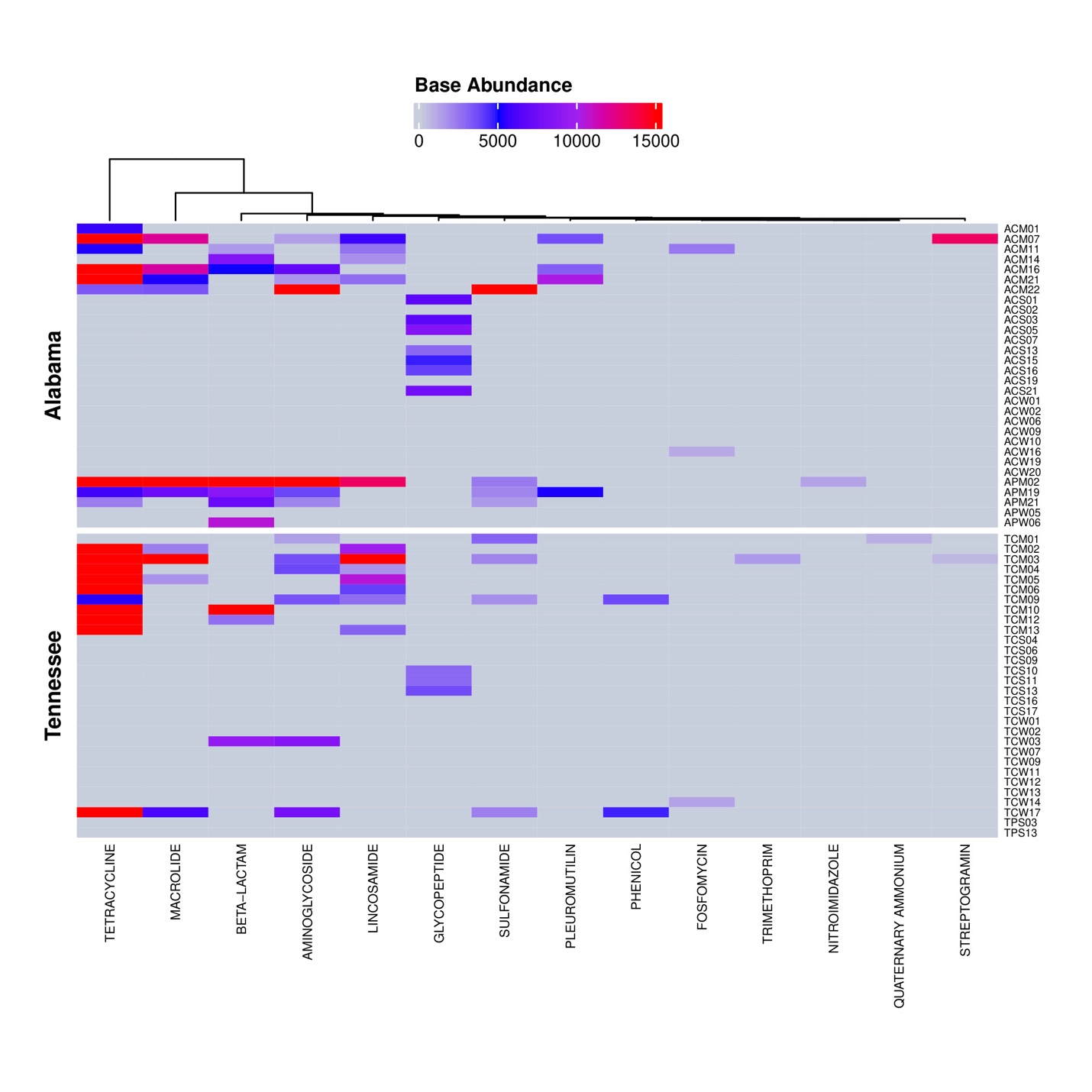


**Supplementary Figure 8:** Antimicrobial resistance profile of microbiota from individual soil, manure and water samples collected from cattle and poultry farming systems in Alabama and Tennessee. For this figure, antimicrobial resistance genes (ARGs) of 70% or higher coverage are considered.


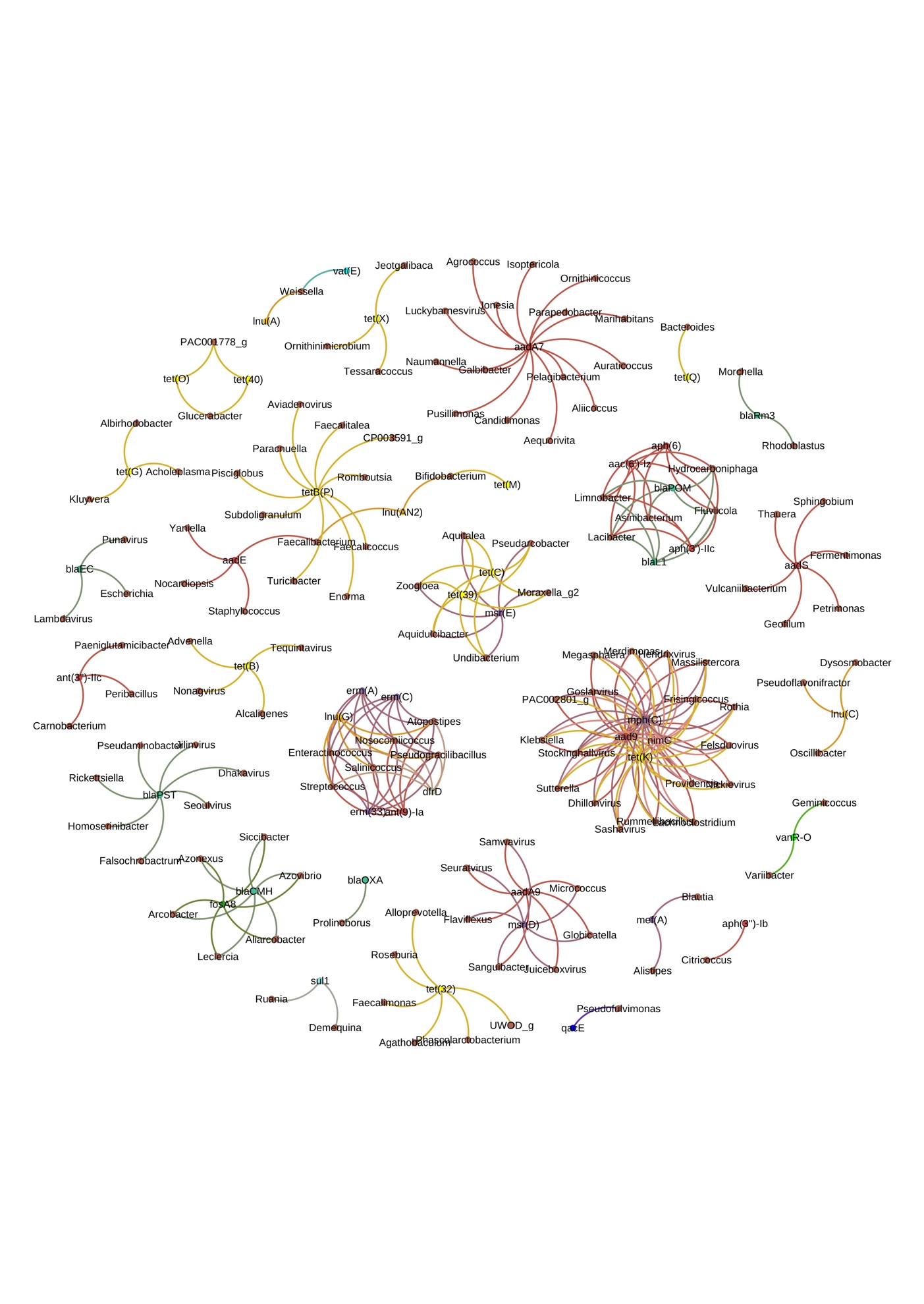


**Supplementary Figure 9:** The network analysis revealing the co-occurrence patterns between antimicrobial resistance gene (ARG) subtypes and microbial taxa. The nodes were colored according to ARG types and genus. A connection represents a strong (Spearman’s correlation coefficient ⍴﹥0.8) and significant (P-value ﹤ 0.01) correlation. Antimicrobial resistance genes (ARGs) with 40% or higher coverage are used to generate this figure.

**2.2** **Supplementary Tables** are attached in the excel sheet.
